# Fluoxetine inhibited the activation of A1 reactive astrocyte in a mouse model of major depressive disorder through astrocytic 5-HT_2B_R/β-arrestin2 pathway

**DOI:** 10.1186/s12974-022-02389-y

**Published:** 2022-01-29

**Authors:** Yinquan Fang, Xiao Ding, Yihe Zhang, Lei Cai, Yuan Ge, Kaiyang Ma, Rong Xu, Shanshan Li, Mengmeng Song, Hong Zhu, Jiaqi Liu, Jianhua Ding, Ming Lu, Gang Hu

**Affiliations:** 1 Jiangsu Key Laboratory of Neurodegeneration, Department of Pharmacology, Nanjing Medical University, 101 Longmian Avenue, Nanjing, 211166 Jiangsu China; 2 Department of Pharmacology, Nanjing University of Chinese Medicine, 138 Xianlin Avenue, Nanjing, 210023 Jiangsu China

**Keywords:** 5-HT_2B_R, Fluoxetine, A1 astrocytes, Major depressive disorder, β-arrestin2

## Abstract

**Background:**

Fluoxetine, a selective serotonin reuptake inhibitor, has been reported to directly bind with 5-HT_2B_ receptor (5-HT_2B_R), but the precise mechanisms, whereby fluoxetine confers the anti-depressive actions via 5-HT_2B_R is not fully understood. Although neuroinflammation-induced A1 astrocytes are involved in neurodegenerative diseases, the role of A1 astrocyte in the pathogenesis and treatment of major depressive disorder (MDD) remains unclear.

**Methods:**

Mice were subjected to chronic mild stress (CMS) for 6 weeks and subsequently treated with fluoxetine for 4 weeks. The depressive-like and anxiety-like behaviors and the activation of A1 reactive astrocyte in hippocampus and cortex of mice were measured. Primary astrocytes were stimulated with A1 cocktail (tumor necrosis factor (TNF)-α, interleukin (IL)-1α and C1q), activated (LPS) microglia-conditioned medium (MCM) or IL-6 for 24 h and the expression of A1-special and A2-special markers were determined using RT-qPCR and western blot. The role of 5-HT_2B_R in the effects of fluoxetine on A1 reactive astrocyte was measured using 5-HT_2B_R inhibitor and siRNA in vitro and AAVs in vivo. The functions of downstream signaling Gq protein and β-arrestins in the effects of fluoxetine on the activation of A1 astrocyte were determined using pharmacological inhibitor and genetic knockout, respectively.

**Results:**

In this study, we found that fluoxetine inhibited the activation of A1 reactive astrocyte and reduced the abnormal behaviors in CMS mice, as well as ameliorated A1 astrocyte reactivity under three different stimulators in primary astrocytes. We also showed that astrocytic 5-HT_2B_R was required in the inhibitory effects of fluoxetine on A1 reactive astrocyte in MDD in vivo and in vitro. We further found that the functions of fluoxetine in the activation of A1 astrocyte were independent of either Gq protein or β-arrestin1 in vitro. β-arrestin2 pathway was the downstream signaling of astrocytic 5-HT_2B_R mediated the inhibitory effects of fluoxetine on A1 astrocyte reactivity in primary astrocytes and CMS mice, as well as the improved roles of fluoxetine in behavioral impairments of CMS mice.

**Conclusions:**

These data demonstrate that fluoxetine restricts reactive A1 astrocyte via astrocytic 5-HT_2B_R/β-arrestin2 pathway in a mouse model of MDD and provide a novel therapeutic avenue for MDD.

**Supplementary Information:**

The online version contains supplementary material available at 10.1186/s12974-022-02389-y.

## Background

Major depressive disorder (MDD) is a heterogeneous psychiatric disorder that affects more than 300 million people worldwide [1], and is one of the leading causes of disability in the world [1, 2]. The symptoms of MDD are recurrent episodes of sadness and despondency accompanied by anhedonia, reduced concentration and energy, memory alterations and recurrent suicidal ideation, which are associated with structural and neurochemical deficits [3–5]. MDD is a multifactorial disease with various causes, including genetic susceptibility, environmental risk factors, stress and other pathological processes, such as inflammation [6–8], but the precise etiopathogenesis of MDD is not fully understood.

Current studies point to astrocyte as a potential target for novel antidepressant drugs associating with their effects on glutamatergic signaling and brain-derived neurotrophic factor levels [9]. Astrocytes are one of most abundant cell populations in the central nervous system (CNS), and play supportive roles in CNS functions, such as neurotransmitter homeostasis, blood–brain barrier integrity and synapse development and plasticity [10, 11]. A variety of CNS diseases can be contributed to astrocyte reactivity [12, 13]. Neuroinflammation and ischemia induced two different reactive astrocyte states, A1 and A2, respectively [14]. A1 reactive astrocytes are induced by activated microglia via secretion of specific cytokines and subsequently amplify inflammatory responses and produce neurotoxicity effects, which are involved in ageing brain and neurodegenerative diseases (NDDs) including Huntington's disease [15], amyotrophic lateral sclerosis [16] and Alzheimer's disease [14]. NLY01, a GLP1R agonist has neuroprotective effects via direct prevention of microglia mediated conversion of astrocytes to an A1 neurotoxic phenotype in models of Parkinson’s disease (PD) [17], indicating that modulation of A1 astrocytes is therapeutic target of PD. Patients with MDD exhibit many features of inflammatory responses, including the increased levels of pro-inflammatory cytokines in peripheral blood, cerebrospinal fluid and brain structures as well as reactive microglia visualized by neuroimaging techniques [18, 19]. Acute stress, such as lipopolysaccharide (LPS) and chronic social defeat stress (CSDS) stress, could induce the generation of A1 astrocytes in mice [20, 21]. Nevertheless, it remains unclear the significance of A1 astrocyte in the pathogenesis and treatment of MDD.

Fluoxetine is one of the major selective serotonin receptor inhibitors (SSRIs) and used as first-line treatment for MDD and other psychiatric disorders [22, 23]. The antidepressant action of fluoxetine is associated with increasing serotonin levels, improving neurogenesis and neuroplasticity as well as reducing inflammatory processes [23–25]. Fluoxetine also could regulate astrocytic functions, such as nutrition support, autophagy and ATP gliotransmission [25–27]. However, nothing is known about the function of fluoxetine in activation of A1 astrocyte. Several studies have found that fluoxetine is also the agonist of 5-HT_2B_ receptor (5-HT_2B_R), a member of the G protein-coupled receptor (GPCR) [28, 29]. The therapeutic effects of fluoxetine on neurotransmission and adult neurogenesis were associated with 5-HT_2B_R [28, 30, 31]. However, the detailed mechanisms of 5-HT_2B_R in the anti-depressive actions of fluoxetine remain to be elucidated.

The purposes of this study are to investigate the effects of fluoxetine on activation of A1 astrocyte in MDD, and elucidate whether its mechanism is relative to 5-HT_2B_R. We found that fluoxetine administration relieved the depressive-like and anxiety-like behaviors in chronic mild stress (CMS) mice models. Fluoxetine also reduced A1 astrocyte reactivity in mice and primary cultured astrocytes. We also demonstrated that the function of fluoxetine in the activation of A1 astrocyte was mediated by astrocytic 5-HT_2B_R and dependent on the downstream β-arrestin2 signaling pathway, while independent of either classical G protein or β-arrestin1 signaling pathway. These findings illustrate that fluoxetine suppresses A1 reactive astrocyte in MDD through astrocytic 5-HT_2B_R/β-arrestin2 pathway, and provide a new strategy for targeting the astrocyte reactivity in the treatment of psychiatric diseases.

## Methods

### Animals

C57BL/6 J WT mice (male, 2 month old and neonatal mice) were purchased from the Animal Core Faculty of Nanjing Medical University. β-arrestin1 knockout (β-arrestin1^−/−^) mice were purchased from the Jackson Laboratory (Las Vegas, NV, USA) and β-arrestin2 knockout (β-arrestin2^−/−^) mice were obtained from Gang Pei’s laboratory (Tongji University, Shanghai, China). β-arrestin1^−/−^ and β-arrestin2^−/−^mice were bred and maintained in the Animal Resource Centre of the Faculty of Medicine, Nanjing Medical University. If not specifically clarified, mice were allowed access to food and water ad libitum and maintained at 22–24 ℃. Light on/off cycles were controlled in a 12/12 h shift. All animal experiments were carried out in compliance with the ethical regulations and approved by Institutional Animal Care and Use Committee of the Nanjing Medical University Experimental Animal Department.

### Injection of adeno-associated virus (AAV)s

The AAV PHP.eB viruses expressing mouse 5-HT_2B_R small interfering RNAs (siRNA, 5-HT_2B_R siRNA1/2, Additional file 1: Table S1) (AAV-mHT_2B_R, 1.76E + 13 v.g./mL) or Control siRNA (AAV-Ctr, 1E + 13v.g./mL) (GeneChem Co, China) under the astrocyte-special gfaABC1D promoter were microinjected bilaterally into the hippocampal CA1 region (AP: − 2.0 mm; ML:1.8 mm; DV:− 1.5 mm) of C57BL/6 J mice (male, 2 month old) at a rate of 0.25 μL/min for 4 min (total 1 μL AAVs) in a stereotaxic apparatus. After 4 weeks, the mice were subjected to CMS to develop a MDD mouse model and administrated with fluoxetine (FLX) described as followed.

### MDD model and drug administration

The CMS model was developed as described previously [30, 32, 33]. Briefly, each mouse was caged singly and adapted to the environment for 3 days. Mice randomly divided into Control + saline, Control + FLX, CMS + saline, CMS + FLX groups (*n* = 8 mice for each group); AAV-Ctrl + Control + saline (*n* = 9), AAV-Ctrl + Control + FLX (*n* = 9), AAV-Ctrl + CMS + saline (n = 11), AAV-Ctrl + CMS + FLX (*n* = 12), AAV-mHT_2B_R + Control + saline (*n* = 9), AAV-mHT_2B_R + Control + FLX (*n* = 9), AAV-mHT_2B_R + CMS + saline (*n* = 12), AAV-mHT_2B_R + CMS + FLX (*n* = 12) groups; WT + Control + saline (*n* = 10), WT + Control + FLX (*n* = 10), WT + CMS + saline (*n* = 10), WT + CMS + FLX (*n* = 11), β-arrestin2^−/−^ + Control + saline (n = 10), β-arrestin2^−/−^ + Control + FLX (n = 9), β-arrestin2^−/−^ + CMS + saline (n = 10), β-arrestin2^−/−^ + CMS + FLX (*n* = 12) groups. Mice were exposed to 2–3 times randomly scheduled, mild stressors, which were not repeated for 3 consecutive days per day for 10 weeks. The mild stressors included cold water (4 °C for 5 min), wet bedding (12 h), the reversal of the light–dark cycle (24 h), empty cage (12 h), restraint (6 h), food and water deprivation (12 h), clipping tails (3 min), strobe light (12 h) and cage tilting (45 °C, 12 h). Between 6 and 10 weeks, mice was injected with normal saline or fluoxetine (10 mg/kg, i.p., Sigma Aldrich, MO, USA) once-daily.

### Behavioral evaluations

#### Sucrose preference test (SPT)

The SPT was performed as described previously [26]. Before test, all mice were acclimated with 1% sucrose solution for 72 h and then deprived of water and food for 24 h, then, one bottle of water and one bottle of 1% sucrose solution were placed in each cage for 12 h. After 6 h, two bottles were exchanged to avoid location effects. The bottles were weighed before and after each test, and the sucrose preference (%) was calculated as followed formula. Sucrose preference (%) = Consumption of sucrose solution / Consumption of sucrose solution and water × 100%.

#### Forced swimming test (FST)

The FST was carried out as described by our previous studies [26, 30, 34]. This test is based on the immobility time when mice are subjected to inescapable conditions [35]. Briefly, mice were individually placed in a 2 L glass cylinder (13 cm diameter × 24 cm height) filled with 14 cm deep water at 22 °C for 6 min. Water was changed every trial. After adaption for 2 min, the behaviors of mice over the next 4 min were recorded by a camera positioned directly in front of the cylinder and analyzed by Force Swim Scan™ software (CleverSys Inc, VA, USA).

#### Tail suspension test (TST)

The TST is a frequently used experiment to assess the antidepressant action [36–38]. Mice were individually suspended from the ceiling of the TST box (50 × 50 × 50 cm) by distal portion of their tails with adhesive tape at approximately 1 cm below the tip of the tails for 6 min. A camera was placed the front of the box and recorded the behavior of mice. The immobility time of tail-suspended mice during the last 4 min were analyzed by Tail suspension Scan™ software (CleverSys Inc).

#### Open field test (OFT)

The OFT is performed to evaluate the mental excitability of mice, which shows an exploratory behavior and a state of tension, fear, and alertness to the new environment [39]. In this test, mice were placed into the center area of each opaque box (50 × 50 × 40 cm) which is divided into a central area (side length: 35 cm) and a border area. The total route of the mice within 10 min was recorded using a camera and the amount of mouse feces (a drop of urine was counted as one pellet) was counted. Before the experiment, the bottom of the box was wiped with alcohol to remove the residual odor that interferes with the behavior of mice. Bouts and duration of mice staying in the central area were analyzed by TopScan Realtime Option (CleverSys Inc).

#### Novelty-suppressed feeding test (NSFT)

The NSFT was performed to evaluate the anxiety of mice [40, 41]. The experimental device and operation are similar with OFT, except that the same number of 6 peanuts with regular shapes are placed in the central area. Mice were put simultaneously into the corners of each box and back to peanuts after fasted for 24 h. We then record the time of mice sniffing peanuts for more than 3 s or the time of chewing peanuts for the first time (The longest record does not exceed 10 min).

#### Social interaction test (SIT)

The SIT is performed to observe the anxiety of mice through detecting the social latency [40, 41]. A perforated plastic box separates placing a social mouse randomly on one side of the opaque box and an empty plastic box on the other. The scope of the social zone is within 2 cm on both sides of the perforated plastic box and 2 cm in front of the box. The corner area is defined as a square area with a corner size of 10 × 10 cm on the opposite side of the box, and the remaining area is a neutral area. Mice were placed in the center area and screened the behavior within 10 min using the camera. Recorded the timepoint when mice started to contact the social mouse for more than 3 s.

### Isolation and treatment of primary cells

Neonatal mice (within 3 days after birth) were used for the isolation of microglia as described elsewhere [42]. Briefly, after stripping the meninges and vessels, the brain tissues were treated with 0.25% trypsin/EDTA (ThermoFisher, USA) and dissociated into single cells. The cells were plated into cell adherent reagent (C1010, Applygen, Beijing, China) pre-coated T75 flasks and cultured in DMEM/F-12 (Gibco, USA), 10% fetal bovine serum (FBS, Gibco) and 1% streptomycin–penicillin mixture (Gibco). The mix glial cells were cultured for 10–14 d and the medium was changed every 3 d. Then flasks were shaken gently at room temperature (RT) to collect the microglia. After centrifugation at 500 g for 10 min, the cells were plated in 24-well plates, incubated in serum-free base medium for 1 h, and treated with lipopolysaccharide (LPS, 100 ng/mL, Sigma Aldrich) for 12 h. The microglia-conditioned medium (MCM) were collected and centrifuged at 12,000 *g* for 10 min at 4 °C and the supernatant was collected to treat astrocytes. About 98% of these cells were positive for ionized calcium binding adapter molecule 1 (Iba1), a marker for microglial cell types.

Primary astrocytes were isolated from WT, β-arrestin1^−/−^ and β-arrestin2^−/−^ neonatal mice (within 3 days after birth) by digested with 0.25% trypsin–EDTA for 5 min as described [43]. The cells were cultured in DMEM (Gibco) medium containing 10% FBS and 1% penicillin–streptomycin mixture. The medium was changed every 3 d. After 7 d, the cells were split onto plates and incubated in serum-free base medium for 1 h. About 98% of these cells were positive for glial fibrillary acidic protein (GFAP), a marker for astrocyte cell types. Cells were pre-treated with PBS or fluoxetine (10 μM) for 2 h as described previously [26]. Then, astrocytes were treated with following stimulators: A1 cocktail containing tumor necrosis factor (TNF)-α (30 ng/mL, 315-01A, Peprotech, USA), interleukin (IL)-1α (3 ng/mL, 211-11A, Peprotech) and C1q (400 ng/mL, 204,876, Millipore, USA), MCMs as mentioned above or IL-6 (100 ng/ml, CA92590, Chemicon, USA) for 24 h. 5-HT_2B_R inhibitor RS-127445 (10 μM, s2698, Selleck) and Gq protein inhibitor YM-254890 (100 nM, QS 3666A, AdipoGen Life Sciences, USA,) were used to pretreat astrocytes for 30 min before fluoxetine administration according to the manufacturer’s instructions and previous report [44].

Fetuses (E15–16) from pregnant mice were used for the preparation of neurons as described previously [45]. Cortices and hippocampus were collected and dissociated with 0.125% trypsin/EDTA. Cells were plated on poly-L-lysine (PLL, 0.05 mg/ml, Sigma, St Louis, MO, USA) -coated 96-well plates at a density of 5 × 10^5^ cells/ml in DMEM/F-12 supplemented with 10% FBS and incubated at 37 °C in 5% CO2 for 6 h. Then, the media were removed and switched to neurobasal media (Gibco) with 2% B27 (Gibco) supplemented with 0.5 mM glutamine. Culture media were changed every 3 days. After 7 days, primary neurons were lysed. About 98% of these cells were positive for microtubule-associated protein 2 (MAP2), a marker for neurons.

### Cell transfection

siRNA targeting 5-HT_2B_R (5-HT_2B_R siRNA1) and a negative Control (NC) siRNA (GenePharma, Shanghai, China) (Additional file 1: Table S1) were transfected into astrocytes using LipofectamineTM RNAi MAX (Invitrogen, Life Technologies, CA, USA) in Opti-MEM reduced serum medium (Gibco) according to the manufacturer’s instructions for 8 h. The mixture was removed and the cells were cultured with normal medium for an additional 40 h (Additional file 2).

### Brain tissue and blood samples processing

For immunohistochemistry (IHC) and immunofluorescence (IF), mice were anesthetized by an intraperitoneal injection of pentobarbital sodium (60 mg/kg, Sigma). After transcardial perfusion with cold PBS and 4% paraformaldehyde (PFA), the brains were harvested, post-fixed with 4% PFA overnight at 4 °C and dehydrated in 20 and 30% sucrose for 1 week. Then brain tissues were snap-frozen and cut by a Leica CM1860 cryostat (20 or 30 μm thick). The brain slices were stored at − 80 °C until use. For real-time reverse transcription polymerase chain reaction (RT-qPCR), western blotting and ELISA, mice were anesthetized and perfused with cold PBS, then the brains were harvested and hippocampus and cortex were collected in Eppendorf tubes and stored at − 80 °C until use. Blood samples were collected from eyeball and allowed to clot for 30 min at RT and overnight at 4 °C. To obtain the serum the blood was centrifuged at 800 g for 30 min at 4 °C and the supernatants were centrifuged at 5000 g for 10 min at 4 °C. The serum were stored at − 80 °C until use.

### IHC

For immunohistochemical analysis [46], frozen 30 μm-thick hippocampal slices were deparaffinized with 3% H_2_O_2_ for 30 min. Nonspecific antibody binding was blocked by 5% bovine serum albumin (BSA, BIOFROXX, Guangzhou, China) in PBS supplemented with 0.3% Triton X-100 for 60 min. Subsequently, the sections were incubated with GFAP (1:1000, Abcam, MA, USA, ab7260) at 4 °C overnight. After washing with PBS, the slides were incubated with goat anti-rabbit antibody (1:1000, Invitrogen) for 60 min at RT. The GFAP positive cells in hippocampal were detected by the DAB staining system (Boster, China), observed under a stereomicroscope (Olympus, Tokyo, Japan) for imaging, and branch number of GFAP-positive cell were calculated by Image J software (National Institutes of Health, Bethesda, Maryland, USA).

### IF

IF staining was carried out as described previously [42]. Frozen 20 μm-thick brain sections were blocked in 5% BSA with 0.3% Triton X-100, and incubated with specific the primary antibodies, including mouse anti-GFAP (1:1000, Millipore, MAB360), rabbit anti-C3 (1:100, Abcam, ab11887), rabbit anti-Serping1 (1:500, Proteintech, IL, USA 12259–1-AP) or rabbit anti-5-HT_2B_R (1:500, SAB Signalway, Nanjing, China, 32964) at 4 °C overnight. The slides were incubated with Alexa 594- or Alexa 555-conjugated secondary antibodies (1:500, Invitrogen) for 1 h and stained with DAPI (Invitrogen), and images were observed by confocal microscopy (Olympus) and analyzed by Image J software. The area of positive signal of GFAP, double positive signal for C3, or Serping1, and GFAP was measured using the interest grayscale threshold analysis with constant settings for minimum and maximum intensities for each staining marker [47–49]. Three brain sections per mouse were used for quantification and the average of three sections was used to represent a data for each mouse.

### RT-qPCR

Total RNA from mouse brain tissues or cultured astrocytes were extracted using Trizol reagent (Invitrogen), and reverse transcribed using HiScript Q RT SuperMix for qPCR Kit (Vazyme Biotech, Nanjing, China) according to the manufacturer’s instructions. The cDNA obtained was mixed with AceQ qPCR SYBR Green Master Mix (High ROX Premixed) (Vazyme Biotech) and gene-specific primers (Additional file 1: Table S1) for real-time PCR in a StepOnePlus instrument (Applied Biosystems, CA, USA). The cycle time values were standardized to GAPDH of the same sample. A relative quantity was calculated using the −ΔΔCT (showed in heatmap) and 2^−ΔΔCT^ (showed in bar chart) methods [17, 50].

### Western blotting

Mouse tissues or primary cultured cells were lysed in RIPA buffer (50 mmol/L Tris.HCl (pH 7.5), 150 mmol/L NaCl, 10% Nonidet P-40 (NP-40), 0.5% Sodium Deoxycholate, 0.1% SDS, and 1 mmol/L PMSF (add before used)) supplemented with protease inhibitor cocktail (Roche, USA). The lysates were centrifuged at 16,000 g for 15 min at 4 °C, and the supernatants were used for immunoblot analysis after determining the protein concentrations by the BCA method (BCA Protein Quantitation Assay, Keygen Biotech, China). The extracts were resolved by SDS/PAGE on an 8–12% gradient separating gel and blotted on PVDF membranes (PALL Gelman, Ann Arbor, MI, USA) using Mini-PROTEAN® Tetra Handcast Systems and Mini Trans-Blot® Cell (Bio-Rad Laboratories, Hercules, CA, USA). Electrophoresis buffer (14.4 g/L Glycine, 3.0 g/L Tris.HCl and 1.0 /L SDS) and transfer buffer(2.93 g/L Glycine, 5.82 g /L Tris.HCl and 200 ml/L Methanol) were used. The membranes were blocked for 1 h at room temperature with 5% fat free milk in Tris-buffered saline Tween-20 (8.0 g/L NaCl, 2.4 g/L Tris base and 1 mL/L Tween-20) and then incubated with the following primary antibodies at 4 °C overnight: rabbit anti-C3 (1:500, ThermoFisher, PA5-21,349), rabbit anti-5-HT_2B_R (1:1000, SAB Signalway, 32,964), rabbit anti-β-arrestin1 (1:1000, Santa Cruz, CA, USA, SC-53780), rabbit anti-β-arrestin2 (1:1000, Cell Signaling Technology, USA, 3857) and mouse anti-β-actin (1:5000, YI FEI XUE Biotechnology, China, YFPA1596). After the membranes were washed three times and incubated with the corresponding HRP-conjugated secondary antibody (1:5000, YI FEI XUE Biotechnology) for 1 h at RT, the bands were detected by enhanced chemiluminescence western blot detection reagents (Pierce) and analyzed with the ImageQuant™ LAS 4000 imaging system (GE Healthcare, USA). The band intensity was determined by densitometry with the aid of the Bio-Rad Gel Doc XR documentation system.

### ELISA

The concentrations of IL-6 in serum, cortex and hippocampus of mice were measured using commercially available ELISA kits (DY406, R&D, USA) according to the manufacturer’s instructions. Serum were diluted to 5 times using Reagent Diluent (1% BSA in PBS, pH 7.2–7.4, 0.2 µm filtered) and the extracts of cortex and hippocampus were diluted to 5 mg/mL using RIPA buffer. Standards were diluted using Reagent Diluent and the final concentrations are 1000, 500, 250, 125, 62.5, 31.25 and 15.625 pg/mL. Coat a 96-well microplate with 100 μL per well of the diluted Capture Antibody and seal the plate overnight at RT. Aspirate each well and wash with Wash Buffer (0.05% Tween® 20 in PBS, pH 7.2–7.4) for three times. After the last wash, remove any remaining Wash Buffer by aspirating or by inverting the plate and blotting it against clean paper towels. Block plates using Reagent Diluent and incubate for 1 h at RT. Repeat the aspiration/wash as above and the plates are ready for sample addition. Add sample or standards (100 μL per well), cover with an adhesive strip and incubate 2 h at RT. Repeat the aspiration/wash as above, and add Detection Antibody (100 μL per well), cover with an adhesive strip and incubate 2 h at RT. Repeat the aspiration/wash as above, and then add working dilution of Streptavidin–HRP (100 μL per well), cover the plate and incubate for 20 min at RT. Avoid placing the plate in direct light and repeat the aspiration/wash as above, and then add Substrate Solution (100 μL per well) and incubate for 20 min at RT. Add Stop Solution (10% H_2_SO_4_, 50 μL per well) and gently tap the plate to ensure thorough mixing. Determine the optical density of each well immediately, using a microplate reader (Thermo) set to 450 nm, with background correction read at 590 nm. A linear standard curve was created according the optical density (O.D.) values and standard concentration. The concentration of the samples was then calculated using the equation of the standard curve and taking into account the dilution of each sample.

### Lactate dehydrogenase (LDH)release assays

Cell viability was determined by measuring the LDH release in the supernatants of astrocytes using a LDH assay kit (Jiancheng Bioengineering technology, Nanjing, China) according to the manufacturer’s instructions.

### Statistical analysis

Statistical tests were carried out using GraphPad Prism 8.0 software. The normal distribution of data was tested using the Shapiro–Wilk test. To compare two groups, Student’s t test was used. To compare multiple groups, two-way repeated-measures ANOVA with Tukey’s post hoc test was applied. Data are presented as the means ± SEM from at least three independent experiments. Differences were considered significant at *P* < 0.05.

## Results

### Chronic fluoxetine administration inhibits A1 astrocyte reactivity in CMS mice models

**Fig. 1:**
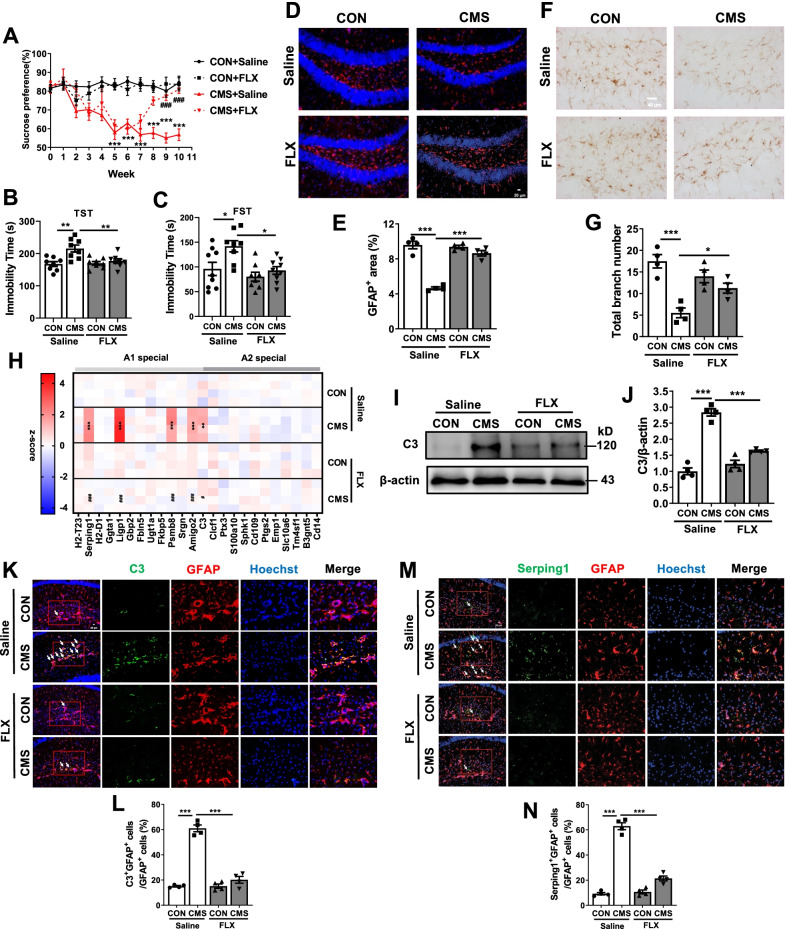
CMS-induced depressive-like behaviors, loss of astrocyte and A1 astrocyte reactivity are rescued by fluoxetine administration. C57BL/6 J mice were subjected to CMS for 6 weeks and then intraperitoneal injected with a vehicle (Control, 0.9% saline) or fluoxetine (10 mg/kg/day) for another 4 weeks. **A** Sucrose preference percentage during 10 weeks. **B**, **C** Immobility time of mice in TST (**B)** and FST (**C**). **D**, **E** Immunofluorescence staining (**D**) and area (**E**) of GFAP^+^ astrocyte in the hippocampus. **F**, **G** Immunohistochemical staining (**F**) and total branch number (**G**) of GFAP^+^ astrocyte in the hippocampus. **H** Heatmap of A1-special and A2-special transcripts in the hippocampus as analyzed by RT-qPCR. **I, J** Protein levels of C3 in the hippocampus. **K**–**N** Proportion of C3 (**K**, **L**) and Serping1 (**M**, **N**) in astrocyte in the hippocampus. Scar bar, 20 μm in **D**, **K**, **M**; Scar bar, 40 μm in **F**. Quantitative data are mean ± s.e. Data were analyzed using two-way ANOVA, then combined with Tukey’s multiple comparison to assess the differences between groups. **A, B**: *n* = 8 per group; **C**: n = 9 CON with saline and CMS with FLX, *n* = 8 CMS with saline and CON with FLX; **D**–**N** *n* = 4 per group; biologically independent animals. **A**, **H**: **P < 0.01, and ***P < 0.001 vs respective Control mice treated with saline; ^*#*^*P* < 0.05, and ^#*##*^*P* < 0.001 vs CMS mice treated with saline. **B**–**G**, **J**, **L**, **N**: *P < 0.05, **P < 0.01, and ***P < 0.001

In this study, C57BL/6 J mice were subjected to the CMS for 6 weeks to establish a mouse model of MDD and followed by chronic fluoxetine treatment for 4 weeks (Additional file 1: Fig. S1A). After CMS stimulation for 6 weeks, the sucrose preference percentage markedly decreased (5–10 weeks: *P* < 0.001), which could be significantly improved by fluoxetine treatment for 3 and 4 weeks (Fig. 1A) (9–10 weeks: *P* < 0.001). Then, their behavioral changes were analyzed by TST, FST, OFT, NSFT and SIT. The immobility time of mice were significantly increased in TST and FST after CMS stimulation (Fig. 1B, C) (TST: *P* < 0.01 and FST: *P* < 0.05). In OFT experiments, the bouts and duration in center were reduced in mice that were exposed to CMS (Additional file 1: Fig. S1B–D) (bouts: *P* < 0.01 and duration: *P* < 0.05). Moreover, the latency time of mice to the first sniff in SIT and to the first feed > 3 s in NSFT were increased after CMS stimulation (Additional file 1: Fig. S1E, F) (*P* < 0.001). All these depressive-like and anxiety-like behaviors induced by CMS were clearly mitigated by chronic fluoxetine administration for 4 weeks (Fig. 1B, C, Additional file 1: Fig. S1B–F) (FST, duration and NSFT: *P* < 0.05; TST and bouts: *P* < 0.01; SIT: *P* < 0.001). Besides, IHC and IF results showed that fluoxetine treatment also reversed the reduction in the number and area of GFAP positive astrocytes in hippocampus of mice induced by CMS stimulation (Fig. 1D–G) (CON + saline vs CMS + saline *P* < 0.001; CMS + saline vs CMS + FLX *P* < 0.05 in IHC; *P* < 0.001 in IF). These data indicated that fluoxetine administration relieved the impairment of astrocyte in CMS mice. Next, we determined the effects of fluoxetine on the types of astrocyte by assessing mRNA levels of A1-specific and A2-specific markers. The transcripts of A1-specific markers (Serping1, Ligp1, Psmb8, Amigo2 and C3) were increased in hippocampus and cortex of CMS mice (hippocampus: *P* < 0.01 in C3; *P* < 0.001 in Serping1, Ligp1, Psmb8 and Amigo2; cortex: *P* < 0.001 in Serping1, Ligp1, Psmb8, Amigo2 and C3), which was reversed by fluoxetine treatment (Fig. 1H, Additional file 1: Fig. S2A) (hippocampus: *P* < 0.05 in C3; *P* < 0.001 in Serping1, Ligp1, Psmb8 and Amigo2; cortex: *P* < 0.001 in Serping1, Ligp1, Psmb8, Amigo2 and C3). However, CMS stimulation and fluoxetine administration failed to affect the levels of A2-specific transcripts (Clcf1, Ptx3, S100a10, Sphk1, Cd109, Ptgs2, Emp1, Slc10a6, Tm4sf1, B3gnt5 and Cd14) in hippocampus and cortex of mice (Fig. 1H, Additional file 1: Fig. S2A). As C3 is one of the characteristics of A1 astrocyte and the levels of C3 reflex the activation of A1 astrocyte[14], we also found that chronic fluoxetine treatment decreased the protein levels of C3 in hippocampus and cortex of CMS mice (Fig. 1I, J, Additional file 1: Fig. S2B, C) (CON + saline vs CMS + saline *P* < 0.01 in cortex; *P* < 0.001 in hippocampus; CMS + saline vs CMS + FLX *P* < 0.001). The enhancement of the percentage of A1 astrocytes, which are C3 and GFAP double-positive (C3^+^GFAP^+^) cells and Serping1 and GFAP double-positive (Serping1^+^GFAP^+^) cells in hippocampus of CMS mice were reduced by chronic fluoxetine treatment (Fig. 1K–N) (*P* < 0.001). These findings suggest that fluoxetine decreases A1 astrocyte reactivity in the CMS mouse model, which might contribute to the antidepressant effects of this drug.

### Fluoxetine inhibits the activation of A1 astrocyte in vitro

**Fig.2:**
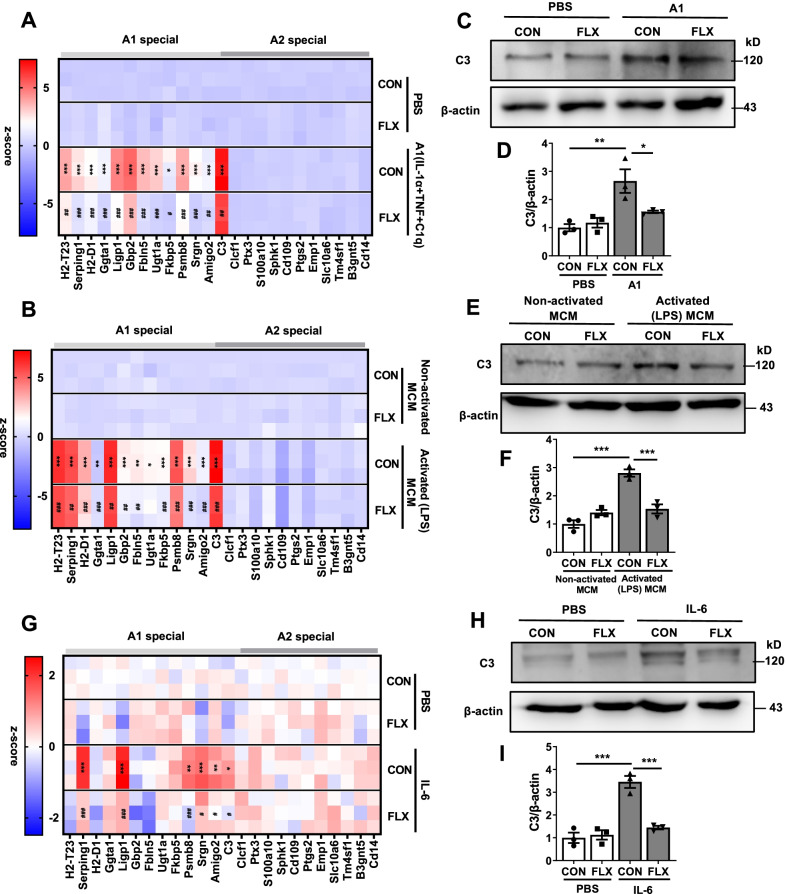
Fluoxetine inhibits the production of A1 reactive astrocytes under three different stimulators.** A**,** B** Heatmap of A1-special and A2-special transcripts in primary astrocytes after A1 cocktail (IL-1α plus TNF-α plus C1q) stimulation (**A**) or activated (LPS)-MCM treatment (**B**) for 24 h as analyzed by RT-qPCR. **C**–**F** Protein levels of C3 in astrocyte after A1 cocktail stimulation (**C**, **D**) or LPS-MCM treatment (**E**,** F**) for 24 h. **G** Heatmap of A1-special and A2-special transcripts in astrocyte after IL-6 stimulation for 24 h as analyzed by RT-qPCR. **H**, **I** Protein levels of C3 in astrocyte after IL-6 stimulation for 24 h. Quantitative data are mean ± s.e. Data were analyzed using two-way ANOVA, then combined with Tukey’s multiple comparison to assess the differences between groups. All data represent the results of three independent experiments. **A**, **B**, **G**: *P < 0.05, **P < 0.01, and ***P < 0.001 vs Control cells treated with PBS (**A**, **G**) or non-activated MCM (**B**); ^*#*^*P* < 0.05, ^*##*^*P* < 0.01, and ^*###*^*P* < 0.001 vs Control cells treated with A1 (**A**), activated (LPS) MCM (**B**) or IL-6 (**G**) stimulation. **D**, **F**, **I**: *P < 0.05, **P < 0.01, and ***P < 0.001

The production of A1 astrocytes were induced by three cytokines together including IL-1α, TNF-α and C1q which were released by activated microglia [14]. To investigate the functions of fluoxetine in the transition of A1 astrocyte in vitro, primary astrocytes (Additional file 1: Fig. S3A) were cultured and treated with A1 cocktail (TNF-α, IL-1α and C1q) or activated (LPS) microglial-conditioned media (LPS-MCM) for 24 h. Compared to control group, fluoxetine treatment reduced the enhancements in the levels of A1-specific mRNA (H2–T23, Serping1, H2–D1, Ggta1, Ligp1, Gbp2, Fbln5, Ugt1a, Fkbp5, Psmb8, Srgn, Amigo2 and C3) induced by A1 cocktail and activated MCM in astrocytes (Fig. 2A, B) (CON + A1 vs CON + PBS *P* < 0.05 in Fkbp5, *P* < 0.001 in other markers; CON + A1 vs FLX + A1 *P* < 0.05 in Fkbp5, *P* < 0.01 in H2–T23, Amigo2 and C3, *P* < 0.001 in other markers; CON + activated (LPS) MCM vs CON + non-activated MCM *P* < 0.05 in Ugt1a, *P* < 0.01 in Ggta1 and Fkbp5; *P* < 0.001 in other markers; CON + activated (LPS) MCM vs FLX + activated (LPS) MCM *P* > 0.05 in Ugt1a, *P* < 0.01 in Serping1, Ligp1, Gbp2, Fbln5, *P* < 0.001 in other markers). While, A1 cocktail or LPS-MCM stimulation as well as fluoxetine administration had no effects on the expression of A2-specific transcripts in primary astrocytes (Fig. 2A, B). In addition, the protein levels of C3 were significantly enhanced in astrocytes after A1 cocktail or LPS-MCM stimulation, which were reversed by fluoxetine treatment (Fig. 2C–F) (CON + A1 vs CON + PBS *P* < 0.01; CON + A1 vs FLX + A1 *P* < 0.05; CON + activated (LPS) MCM vs CON + non-activated MCM, and CON + activated (LPS) MCM vs FLX + activated (LPS) MCM *P* < 0.001). Furthermore, LDH release in supernatant was measured and the results showed that fluoxetine decreased the death of astrocyte induced by A1 cocktail and LPS-MCM stimulation (Additional file 1: Fig. S3B, C) (CON + A1 vs CON + PBS, CON + A1 vs FLX + A1, and CON + activated (LPS) MCM vs CON + non-activated MCM *P* < 0.001; CON + activated (LPS) MCM vs FLX + activated (LPS) MCM *P* < 0.05). Meta-analysis of adult, adolescent and children studies found a positive association between depressive symptoms and IL-6, a pleiotropic cytokine with roles in immunity [51, 52]. We showed that the levels of IL-6 were significantly increased in serum, cortex and hippocampus of mice after CMS stimulation, which were attenuated by fluoxetine treatment (Additional file 1: Fig. S2D) (CON + saline vs CMS + saline *P* < 0.05 in hippocampus, *P* < 0.001 in serum and cortex; CMS + saline vs CMS + FLX *P* < 0.05 in hippocampus and cortex, *P* < 0.001 in serum). We also found that although the mRNA levels of A2-special markers did not change, A1-specific markers (Serping1, Ligp1, Psmb8, Srgn, Amigo2 and C3) were increased in astrocytes after IL-6 stimulation (Fig. 2G) (*P* < 0.05 in C3; *P* < 0.01 in Psmb8 and Amigo2; *P* < 0.001 in Serping1, Ligp1 and Srgn). Moreover, the expression of C3 protein and release of LDH were also enhanced in astrocytes after IL-6 stimulation (Fig. 2H–I, Fig. S3D) (*P* < 0.001). Fluoxetine treatment significantly blocked the activation of A1 astrocyte and release of LDH induced by IL-6 (Fig. 2G–I, Additional file 1: Fig. S3D) (*P* < 0.05 in C3, Srgn and Amigo2; *P* < 0.01 in LDH; *P* < 0.001 in Serping1, Ligp1 and Psmb8 and C3 protein). These data above indicate that fluoxetine inhibits the activation of A1 astrocyte under three different stimulation in vitro.

### The astrocytic 5-HT_2B_R is involved the inhibitory effects of fluoxetine on the activation of A1 astrocyte in vitro and in vivo

**Fig.3:**
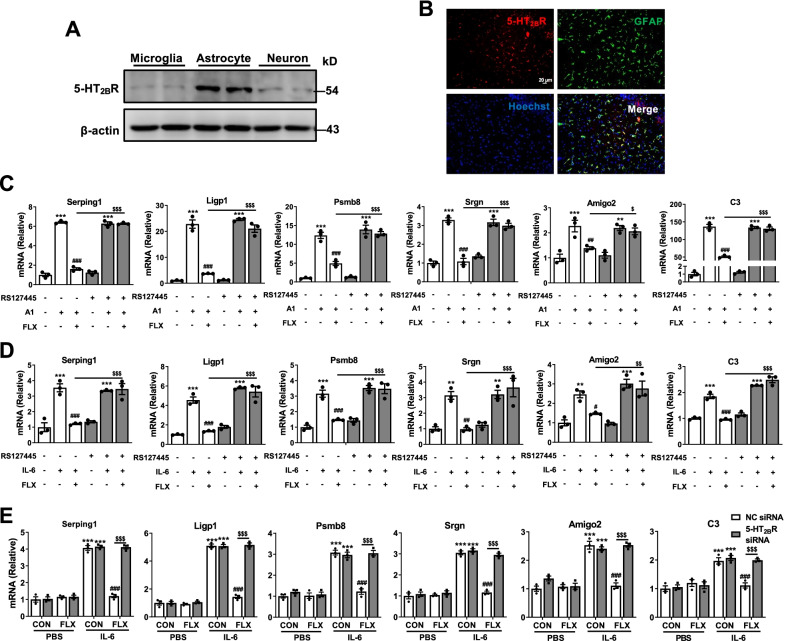
**5**-HT_2B_R is required for the effects of fluoxetine on activation of A1 astrocyte in vitro.** A** Expression of 5-HT_2B_R on primary cultured microglia, astrocyte and neuron. **B** Expression of 5-HT_2B_R on astrocyte in the hippocampus. Similar results were obtained in 3 separate experiments. **C**, **D** Effects of 5-HT_2B_R inhibitor, RS127445, on the expression of A1-special transcripts in astrocytes with or without fluoxetine treatment after A1 cocktail **C** or IL-6 **D** stimulation for 24 h. **E** Effects of 5-HT_2B_R knockdown on the mRNA levels of A1-special markers in astrocytes with or without fluoxetine treatment after IL-6 stimulation for 24 h. Scale bars, 20 µm in **B**. Quantitative data are mean ± s.e. Data were analyzed using two-way ANOVA, then combined with Tukey’s multiple comparison to assess the differences between groups. All data represent the results of three independent experiments. **C**–**E**: **P < 0.01, and ***P < 0.001 vs respective Control cells treated with PBS; ^#^*P* < 0.05, ^##^*P* < 0.01, and ^###^*P* < 0.001 vs Control cells treated with A1 (**C**) or IL-6 (**D, E**) stimulation; ^$^*P* < 0.05, ^$$^*P* < 0.01, and ^$$$^*P* < 0.001

Previous study implied that fluoxetine has relatively high affinity with 5-HT_2B_R in astrocytes [28]. In this study, we found higher concentrations of 5-HT_2B_R proteins in primary cultured astrocytes than in primary cultured microglia and neurons (Fig. 3A, Additional file 1: Fig. S3A). Immunofluorescent imaging also showed that 5-HT_2B_R was mainly colocalized with GFAP positive astrocytes in the hippocampus of mice (Fig. 3B). Therefore, we used 5-HT_2B_R inhibitor RS-127445 to investigate whether 5-HT_2B_R participate in the effects of fluoxetine on the A1 astrocyte reactivity in vitro. As shown in Fig. 3C, D, RS-127445 had no effect on the mRNA levels of A1-specific markers (Serping1, Ligp1, Psmb8, Srgn, Amigo2 and C3) in astrocytes after A1 cocktail or IL-6 stimulation, while blocked the inhibitory effects of fluoxetine on the transcripts of A1-specific markers (CON + A1 vs CON + PBS *P* < 0.001; CON + A1 + RS-127445 vs CON + PBS + RS-127445 *P* < 0.01 in Amigo2; *P* < 0.001 in other markers; CON + A1 vs FLX + A1 *P* < 0.01 in Amigo2; *P* < 0.001 in other markers; FLX + A1 vs FLX + A1 + RS-127445 *P* < 0.05 in Amigo2; *P* < 0.001 in other markers; CON + IL-6 vs CON + PBS *P* < 0.01 in Srgn and Amigo2; *P* < 0.001 in other markers; CON + IL-6 + RS-127445 vs CON + PBS + RS-127445 *P* < 0.01 in Srgn; *P* < 0.001 in other markers; CON + IL-6 vs FLX + IL-6 *P* < 0.05 in Amigo2; *P* < 0.01 in Srgn; *P* < 0.001 in other markers; FLX + IL-6 vs FLX + IL-6 + RS-127445 *P* < 0.01 in Amigo2; *P* < 0.001 in other markers). Next, the expression of 5-HT_2B_R was reduced by siRNA (Additional file 1: Fig. S4) and the functions of fluoxetine in the A1 astrocyte reactivity were determined using RT-qPCR. Fluoxetine treatment decreased the activation of A1 astrocyte induced by IL-6 (*P* < 0.001), which was reversed by 5-HT_2B_R knockdown in astrocytes (Fig. 3E) (*P* < 0.001). These results suggest that 5-HT_2B_R contributes to the inhibitory roles of fluoxetine in the A1 astrocyte reactivity in vitro.

**Fig. 4:**
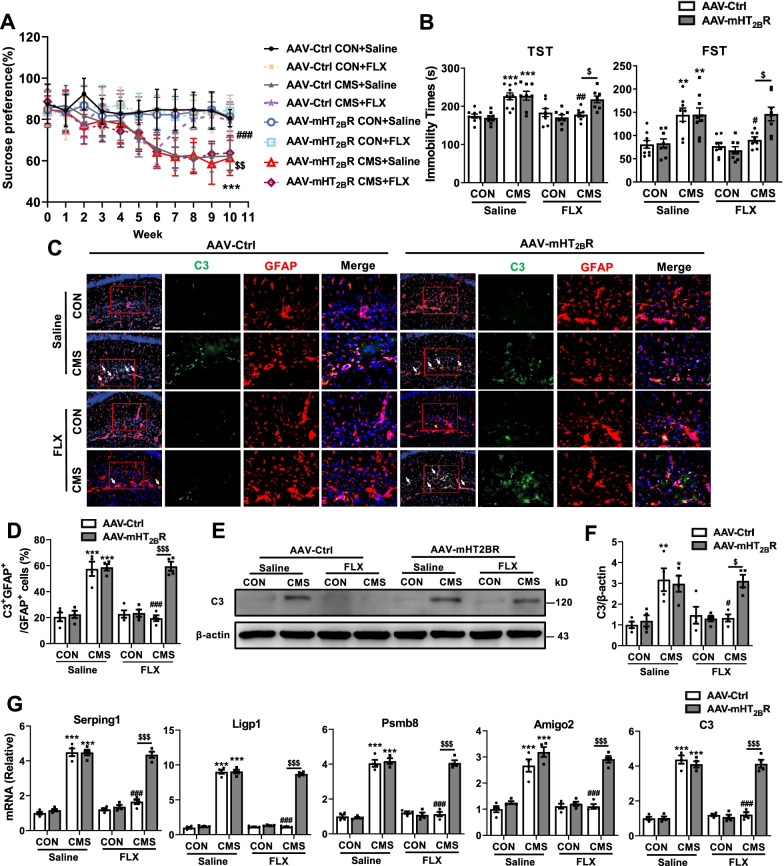
Astrocytic 5-HT_2B_R knockdown blocks effects of fluoxetine on CMS-induced depressive-like behaviors and A1 astrocyte reactivity. **A** Sucrose preference percentage of mice during 10 week treatment after AAV injection for 4 weeks. **B** Immobility time of mice in TST and FST. **C**, **D** Expression of C3 on astrocyte in the hippocampus DG of CON and CMS mice after AAV injection. **E**, **F** Protein levels of C3 in the hippocampus. **G** The levels of A1-special genes in the hippocampus. Scar bar, 20 μm in **C**. Quantitative data are mean ± s.e. Data were analyzed using two-way ANOVA, then combined with Tukey’s multiple comparison to assess the differences between groups. **A**, **B**: n = 9 CON with Control AAV and saline; n = 8 (**B**), *n* = 9 (**A**) CON with Control AAV and FLX; *n* = 8 (**B**-FST), n = 10 (**A**, **B**-TST) CMS with Control AAV and saline; n = 8 (**B**), *n* = 10 (**A**) CMS with Control AAV and FLX; *n* = 8 CON with mHT_2B_R AAV and saline; n = 8 CON with mHT_2B_R AAV and FLX; n = 8 (**B**-TST), *n* = 9 (**A**, **B**-FST) CMS with mHT_2B_R AAV and saline; *n* = 8 CMS with mHT_2B_R AAV and FLX; **C**–**G**: *n* = 4 per group; biologically independent animals. **A**: ***P < 0.001 vs respective Control mice treated with saline; ^*###*^*P* < 0.001 vs CMS mice injected with Control AAV and treated with saline; ^*$$*^*P* < 0.01vs CMS mice injected with Control AAV and treated with fluoxetine; **B**, **D**, **F** and **G**: *P < 0.05, **P < 0.01, and ***P < 0.001 vs respective Control mice treated with saline; ^*#*^*P* < 0.05, ^*##*^*P* < 0.01, and ^*###*^*P* < 0.001 vs CMS mice injected with Control AAV and treated with saline; ^*$*^*P* < 0.05, and ^*$$$*^*P* < 0.001

We further elucidated the function of astrocytic 5-HT_2B_R in the effects of fluoxetine on A1 astrocyte reactivity in vivo. AAVs carrying the astrocyte-specific promoter gfaABC1D (Additional file 1: Fig. S5A) were used to knock down 5-HT_2B_R in astrocyte of hippocampus as confirmed by western blot and immunostaining (Additional file 1: Fig. S5B, C). After AAVs injection in the hippocampus for 4 weeks, the mice were subjected to CMS for 6 weeks and subsequently administrated with fluoxetine for 4 weeks, and then behavioral tests were measured (Additional file 1: Fig. S5D). For depressive-like behaviors, the improved functions of fluoxetine in SPT,TST and FST of CMS mice were significantly reduced by AAV-mediated knockdown of astrocytic 5-HT_2B_R (Fig. 4A, B) (AAV-Ctrl + CMS + Saline vs AAV-Ctrl + CON + Saline, and AAV-mHT_2B_R + CMS + Saline vs AAV-mHT_2B_R + CON + Saline *P* < 0.01 in FST; *P* < 0.001 in SPT and TST; AAV-Ctrl + CMS + Saline vs AAV-Ctrl + CMS + FLX *P* < 0.05 in FST; *P* < 0.01 in TST and *P* < 0.001 in SPT; AAV-mHT_2B_R + CMS + FLX vs AAV-Ctrl + CMS + FLX *P* < 0.05 in TST and FST; *P* < 0.01 in SPT). For anxiety-like behaviors (OFT, SIT and NSFT), the attenuated roles of fluoxetine in CMS mice were also blocked by 5-HT_2B_R knockdown in astrocytes (Additional file 1: Fig. S6A–E) (AAV-Ctrl + CMS + Saline vs AAV-Ctrl + CON + Saline, and AAV-Ctrl + CMS + Saline vs AAV-Ctrl + CMS + FLX *P* < 0.05 in NSFT; *P* < 0.001 in Bouts, Duration and SIT; AAV-mHT_2B_R + CMS + Saline vs AAV-mHT_2B_R + CON + Saline *P* < 0.001; AAV-mHT_2B_R + CMS + FLX vs AAV-Ctrl + CMS + FLX *P* < 0.05 in Bouts; *P* < 0.01 in Duration and NSFT; *P* < 0.001 in SIT). Next, we determine A1 astrocyte reactivity in mice using immunostaining, western blot and RT-qPCR. As shown in Fig. 4C–F, the percentage of C3^+^GFAP^+^ A1 astrocytes and the protein levels of C3 in hippocampus of CMS mice were markedly decreased by fluoxetine treatment (AAV-Ctrl + CMS + Saline vs AAV-Ctrl + CON + Saline *P* < 0.01 in western blot; *P* < 0.001 in IF; AAV-mHT_2B_R + CMS + Saline vs AAV-mHT_2B_R + CON + Saline, and AAV-Ctrl + CMS + Saline vs AAV-Ctrl + CMS + FLX *P* < 0.05 in western blot; *P* < 0.001 in IF), which were restored by astrocytic 5-HT_2B_R knockdown (*P* < 0.05 in western blot; *P* < 0.001 in IF). In addition, the inhibitory effects of fluoxetine on the levels of A1-specific transcripts (Serping1, Ligp1, Psmb8, Amigo2 and C3) in the hippocampus and cortex of CMS mice were blocked by AAV-mediated astrocytic 5-HT_2B_R knockdown (Fig. 4G, Additional file 1: Fig. S6F) (*P* < 0.001). These data indicate that astrocytic 5-HT_2B_R is required for the effects of fluoxetine on behavioral impairments and A1 astrocyte reactivity in CMS mice.

### The repressed function of fluoxetine in the A1 astrocyte reactivity is independent of classical G protein pathway

**Fig. 5:**
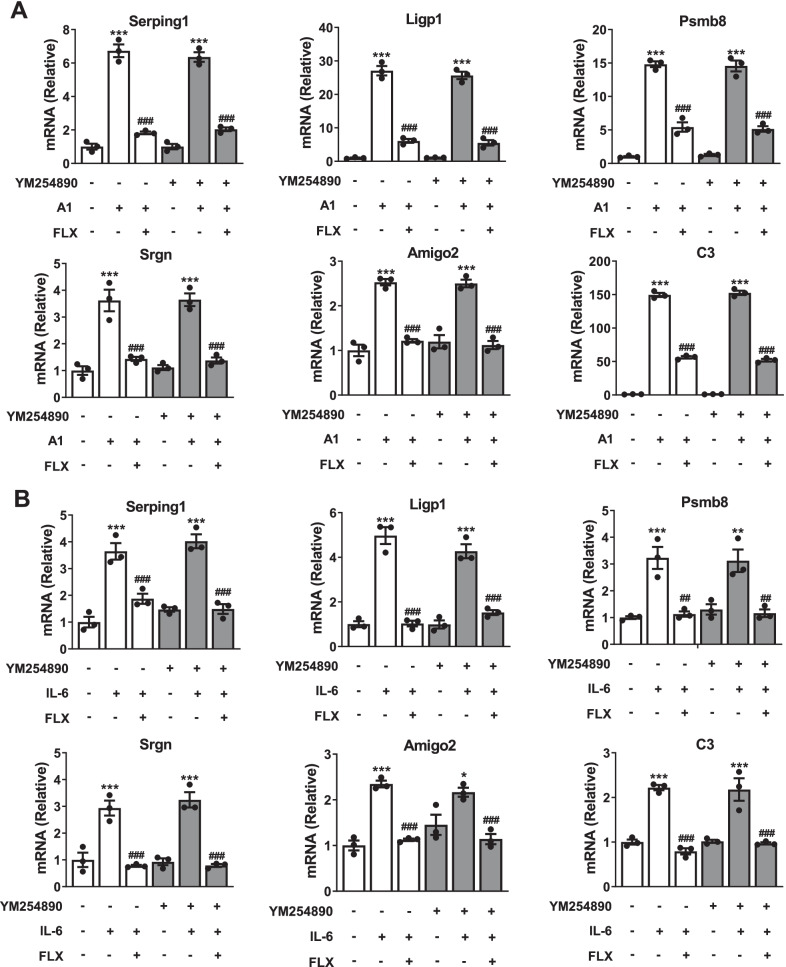
Functions of fluoxetine in A1 astrocyte reactivity are independent of classical G protein pathways. **A, B** Effects of the inhibitor of Gq protein, YM-254890 on the mRNA levels of A1-special markers in astrocytes with or without fluoxetine treatment after A1 (**A**) or IL-6 (**B**) stimulation. Quantitative data are mean ± s.e. Data were analyzed using two-way ANOVA, then combined with Tukey’s multiple comparison to assess the differences between groups. All data represent the results of three independent experiments. **P < 0.01, and ***P < 0.001 vs respective Control cells treated with PBS; ^*##*^*P* < 0.01, and ^*###*^*P* < 0.001 vs Control cells treated with A1 (**A**) or IL-6 (**B**) stimulation

Activation of 5-HT_2B_R stimulates classical Gq protein pathways and β-arrestins-dependent signaling, leading to diverse functions [53, 54]. We explored the possible roles of these two signaling pathways in the effects of fluoxetine on A1 astrocyte reactivity. First, to measure the role of the classical G protein pathway, YM254890, an Gq protein inhibitor was used. As shown in Fig. 5, the enhancement in the mRNA levels of A1-special markers induced by either A1 cocktail or IL-6 stimulation were decreased by fluoxetine treatment, and YM254890 failed to affect the above effects of fluoxetine (CON + A1 vs CON + PBS, CON + A1 + YM254890 vs CON + PBS + YM254890, CON + A1 vs FLX + A1, and CON + A1 + YM254890 vs FLX + A1 + YM254890 *P* < 0.001; FLX + A1 + YM254890 vs FLX + A1: *P* > 0.05; CON + IL-6 vs CON + PBS *P* < 0.001; CON + IL-6 + YM254890 vs CON + PBS + YM254890 *P* < 0.05 in Amigo2; *P* < 0.01 in Psmb8; *P* < 0.001 in other markers; CON + IL-6 vs FLX + IL-6, and CON + IL-6 + YM254890 vs FLX + IL-6 + YM254890 *P* < 0.01 in Psmb8; *P* < 0.001 in other markers; FLX + IL-6 + YM254890 vs FLX + IL-6: *P* > 0.05). The data indicate that the classical G protein pathway isn’t the downstream signaling of astrocytic 5-HT_2B_R regulated the inhibitory functions of fluoxetine in A1 astrocyte activation.

### Inhibitory effects of fluoxetine on the A1 astrocyte reactivity are dependent on β-arrestin2-mediated pathway

**Fig. 6:**
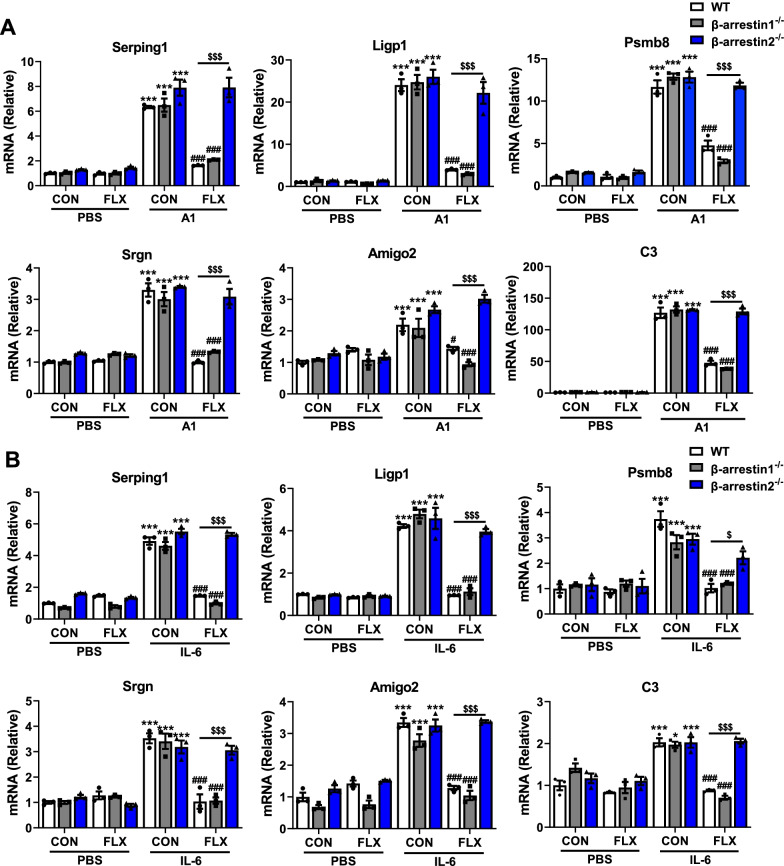
β-arrestin 2, rather than β-arrestin 1 participates in the effects of fluoxetine on A1 reactive astrocytes.** A**, **B** Effects of β-arrestin 1 or β-arrestin 2 knockout on the levels of A1-special genes in astrocytes with or without fluoxetine treatment after A1 cocktail (**A**) or IL-6 (**B**) stimulation. Quantitative data are mean ± s.e. Data were analyzed using two-way ANOVA, then combined with Tukey’s multiple comparison to assess the differences between groups. All data represent the results of three independent experiments. *P < 0.05, and ***P < 0.001 vs respective Control cells treated with PBS; ^*#*^*P* < 0.05, and ^*###*^*P* < 0.001 vs WT Control cells treated with A1 (**A**) or IL-6 (**B**) stimulation; ^*$*^*P* < 0.05, and ^*$$$*^*P* < 0.001

β-arrestins have two isoforms, β-arrestin1 and β-arrestin2, which regulate diverse physiological and pathophysiological processes [55]. We explored the roles of β-arrestin1 and β-arrestin2 in the inhibitory effects of fluoxetine on the activation of A1 reactive astrocyte by knocking out them separately. There were no changes in the mRNA expressions of A1 markers between WT and β-arrestin1-deficient (Additional file 1: Fig. S7A) astrocytes after fluoxetine treatment with or without A1 cocktail and IL-6 stimulation (Fig. 6) (WT CON + A1 vs WT CON + PBS, β-arrestin1^−/−^ CON + A1 vs β-arrestin1^−/−^ CON + PBS, and β-arrestin1^−/−^ FLX + A1 vs β-arrestin1^−/−^ CON + A1 *P* < 0.001; WT FLX + A1 vs WT CON + A1 *P* < 0.05 in Amigo2; *P* < 0.001 in other markers; β-arrestin1^−/−^ FLX + A1 vs WT FLX + A1 *P* > 0.05; WT CON + IL-6 vs WT CON + PBS, WT FLX + IL-6 vs WT CON + IL-6, and β-arrestin1^−/−^ FLX + IL-6 vs β-arrestin1^−/−^ CON + IL-6 *P* < 0.001; β-arrestin1^−/−^ CON + IL-6 vs β-arrestin1^−/−^ CON + PBS *P* < 0.05 in C3; *P* < 0.001 in other markers; β-arrestin1^−/−^ FLX + IL-6 vs WT FLX + IL-6 *P* > 0.05). Whereas, ablation of β-arrestin2 (Additional file 1: Fig. S7B) significantly abolished the ameliorative actions of fluoxetine on the transcript levels of A1 markers in astrocytes after A1 cocktail, IL-6 and activated MCM stimulation (Fig. 6, Additional file 1: Fig. S7C) (β-arrestin2^−/−^ CON + A1 vs β-arrestin2^−/−^ CON + PBS, β-arrestin2^−/−^ FLX + A1 vs WT FLX + A1, and β-arrestin2^−/−^ CON + IL-6 vs β-arrestin2^−/−^ CON + PBS *P* < 0.001; β-arrestin2^−/−^ FLX + IL-6 vs WT FLX + IL-6 *P* < 0.05 in Psmb8; *P* < 0.001 in other markers; WT CON + activated (LPS) MCM vs WT CON + non-activated MCM, and β-arrestin2^−/−^ CON + activated (LPS) MCM vs β-arrestin2^−/−^ CON + non-activated MCM *P* < 0.001; WT FLX + activated (LPS) MCM vs WT CON + activated (LPS) MCM *P* < 0.01 in C3; *P* < 0.001 in other markers; β-arrestin2^−/−^ FLX + activated (LPS) MCM vs WT FLX + activated (LPS) MCM *P* < 0.01 in Amigo2 and C3; *P* < 0.001 in other markers). These results suggest that β-arrestin2, rather than β-arrestin1 is involved in the inhibitory effects of fluoxetine on activation of A1 astrocyte in vitro.

**Fig.7:**
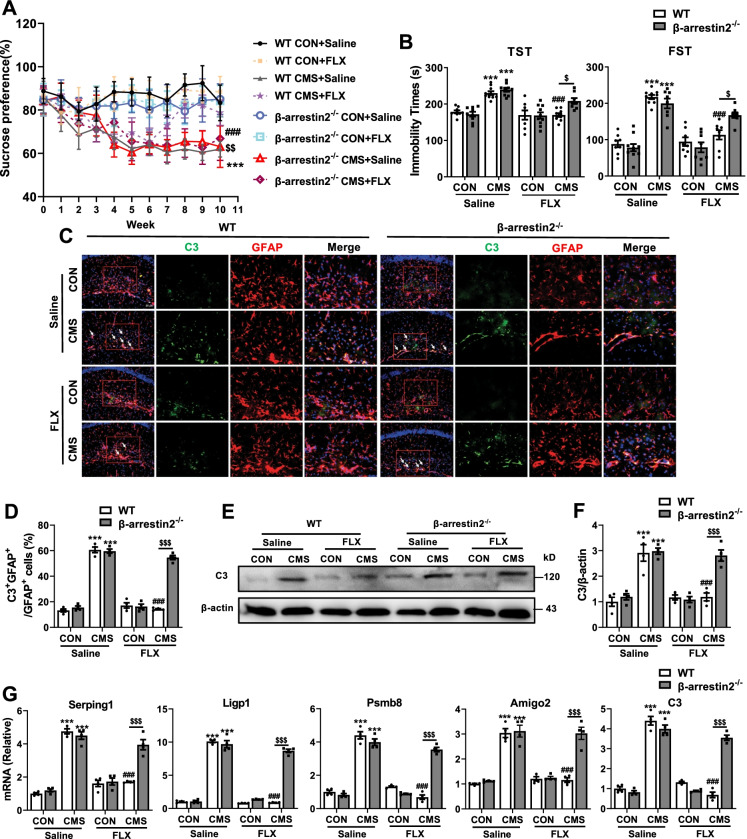
β-arrestin 2 depletion reverses roles of fluoxetine in depressive-like behaviors and A1 astrocyte reactivity in CMS mice.** A** Sucrose preference percentage of WT and β-arrestin 2 knockout mice during 10 week treatment. **B** Immobility time of mice in TST and FST. **C**, **D** Expression of C3 on astrocyte in the hippocampus of WT and β-arrestin 2^*−/−*^ mice with or without fluoxetine treatment. **E**, **F** Protein levels of C3 in the hippocampus. **G** Levels of A1-special genes in the hippocampus. Scar bar, 20 μm in **C**. Quantitative data are mean ± s.e. Data were analyzed using two-way ANOVA, then combined with Tukey’s multiple comparison to assess the differences between groups. **A**, **B**: n = 8 (**A**, **B-**FST), *n* = 9 (**B-**TST) WT CON with saline; n = 8 WT CON with FLX; *n* = 8 (**A**), *n* = 10 (**B**) WT CMS with saline; *n* = 8 (**B**-FST), *n* = 9 (**A**, **B**-TST) WT CMS with FLX; n = 8 (**A**), *n* = 9 (**B-**TST), *n* = 10 (**B-**FST) β-arrestin 2^−/−^ CON with saline; *n* = 8 (**A**, **B-**FST), n = 10 (**B-**TST) β-arrestin 2^−/−^ CON with FLX; *n* = 8 (**A**, **B-**FST), n = 9 (**B-**TST) β-arrestin 2^−/−^ CMS with saline; n = 8 β-arrestin 2^−/−^ CMS with FLX; **C**-**G**: n = 4 per group; biologically independent animals. **A**: ***P < 0.001 vs respective Control mice treated with saline; ^*###*^*P* < 0.001 vs WT CMS mice treated with saline; ^*$$*^*P* < 0.01vs WT CMS mice treated with fluoxetine; **B**, **D**, **F** and **G**: ***P < 0.001 vs respective Control mice treated with saline; ^*###*^*P* < 0.001 vs WT CMS mice treated with saline; ^*$*^*P* < 0.05, ^*$$$*^*P* < 0.001

Next, we further elucidated the role of β-arrestin2 in the effects of fluoxetine on activation of A1 astrocyte in MDD in vivo. Chronic fluoxetine treatment improved depressive-like behaviors, including decreased sucrose preference of SPT and increased immobility time of TST and FST in CMS mice, which were reversed by β-arrestin2 knockout (Fig. 7A, B) (WT CMS + Saline vs WT CON + Saline, β-arrestin2^−/−^ CMS + saline vs β-arrestin2^−/−^ CON + Saline, and WT CMS + FLX vs WT CMS + Saline *P* < 0.001; β-arrestin2^−/−^ CMS + FLX vs WT CMS + FLX *P* < 0.05 in TST and FST; *P* < 0.01 in SPT). In addition, β-arrestin2 deficient blocked the improved effects of fluoxetine on anxiety-like behaviors, including increased bouts and duration in center of OFT (Additional file 1: Fig. S8A–C) (WT CMS + Saline vs WT CON + Saline, and β-arrestin2^−/−^ CMS + saline vs β-arrestin2^−/−^ CON + Saline *P* < 0.001; WT CMS + FLX vs WT CMS + Saline, and β-arrestin2^−/−^ CMS + FLX vs WT CMS + FLX *P* < 0.05 in duration; *P* < 0.001 in bouts) and decreased latency time of mice to the first sniff in SIT and to the first feed > 3 s in NSFT after CMS stimulation (Additional file 1: Fig. S8D, E) (WT CMS + Saline vs WT CON + Saline, β-arrestin2^−/−^ CMS + saline vs β-arrestin2^−/−^ CON + Saline, and WT CMS + FLX vs WT CMS + Saline *P* < 0.001; β-arrestin2^−/−^ CMS + FLX vs WT CMS + FLX *P* < 0.01 in SIT; *P* < 0.001 in NSFT). We then assessed the activation of A1 astrocyte induced by CMS stimulation in WT and β-arrestin2 knockout mice with or without fluoxetine treatment. Immunostaining results showed that the proportion of A1 astrocytes in the hippocampus of CMS mice were reduced by chronic fluoxetine administration, which were abolished by β-arrestin2 knockout (Fig. 7C, D) (*P* < 0.001). Moreover, ablation of β-arrestin2 reversed the inhibitory roles of fluoxetine in the levels of C3 protein in hippocampus of mice after CMS stimulation (Fig. 7E, F) (*P* < 0.001). Furthermore, the ameliorative effects of fluoxetine on the transcript levels of A1 markers in hippocampus and cortex of CMS mice were blocked by β-arrestin2 deficient (Fig. 7G, Additional file 1: Fig. S8F) (*P* < 0.001). These data suggest that β-arrestin2 participates in the functions of fluoxetine in behavioral impairments and A1 astrocyte reactivity in CMS mouse model. Collectively, β-arrestin2 pathway is a downstream effector of 5-HT_2B_R mediated the inhibitory effects of fluoxetine on A1 astrocyte reactivity in MDD in vitro and in vivo.

## Discussion

In this paper, we demonstrate that fluoxetine inhibits the activation of A1 astrocyte in MDD through 5-HT_2B_R/β-arrestin2 pathway in vivo and in vitro (Additional file 1: Fig. S9). From the results presented above, two points are particularly noteworthy. First, our study confirmed that fluoxetine inhibits A1 astrocyte reactivity both in CMS mice and primary cultured astrocytes. In many NDDs, A1 astrocytes not only have potently neurotoxic effects, but they also amplify inflammatory microglial responses [14, 17, 56]. Several postmortem studies found that reduced astrocyte densities may be related to depressive episodes, and elevated astrocyte protein S100 beta in cerebrospinal fluid serum could be a biomarker of MDD [57]. Recently studies implied that appearance of A1 astrocytes is associated with depression-like behaviors induced by LPS and acute stress [20, 58]. Our in vivo data showed that fluoxetine reduced the activation of A1 astrocyte and the loss of astrocyte as well as ameliorated depressive-like and anxiety-like behaviors in CMS mice models. Moreover, our in vitro results showed that in addition to activated MCM and A1 cocktail, IL-6, a kind of cytokine that positively associated with MDD, caused higher transcript levels of A1 markers and LDH secretion in primary astrocytes, suggesting that the development of MDD is associated with the activation of A1 astrocyte. Fluoxetine treatment blocked the activation of A1 reactive astrocyte after those three kinds of stimulator. Our study indicate that the reduction of A1 astrocyte reactivity are involved the antidepressant effects of fluoxetine in CMS mouse model and A1 phenotype regulators are likely to be therapeutic interventions of MDD.

Second, we also verified that the astrocytic 5-HT_2B_R/β-arrestin2 pathway was critically involved in the effects of fluoxetine on activation of A1 astrocyte and abnormal behaviors in MDD. 5-HT_2B_R is one of the serotonin receptor subtypes that modulate various CNS function, such as sleep–wake cycles, emesis, appetite, mood, memory, breathing and cognition [59, 60]. In addition, the *Htr2b* gene, which encodes the 5-HT_2B_R is expressed in neonatal microglia. Ablation of this gene results in overexpression of several cytokine receptors genes under basal conditions as well as an increasing in neuroinflammatory responses upon LPS stimulation in neonatal microglia [61, 62]. Consistent with previous studies [29], we found that astrocytes expressed high levels of 5-HT_2B_R in the hippocampus of mice. Recent studies have highlighted that the expression of astrocytic 5-HT_2B_R is associated with development and treatment of MDD [63, 64]. The present data showed that both gene knockdown and pharmacological inhibitor of 5-HT_2B_R reversed the effects of fluoxetine on activation of A1 astrocyte after either A1 cocktail or IL-6 stimulation. AAV-mediated astrocytic 5-HT_2B_R deficiency blocked the improved actions of fluoxetine on behavioral impairments and A1 astrocyte reactivity in hippocampus of CMS mice. The present observations together suggest that 5-HT_2B_R in astrocyte is involved anti-depressive roles of fluoxetine.

5-HT_2B_R is a member of the GPCR family, which is the largest family of membrane protein receptors, and is the target for many drugs in treating a wide range of diseases [65]. Several studies have implied the relationship between antidepressant effects of SSRIs and 5-HT_2B_R. For example, activation of ERK1/2 pathway induced by fluoxetine is abolished by 5-HT_2B_R inhibitors and siRNA-mediated 5-HT_2B_R deletion [66]. Another paper describes leptin enhancing the anti-depressive behaviors of fluoxetine after sleep deprivation via increasing the levels of astrocytic 5-HT_2B_R [64]. However, few detailed mechanisms of 5-HT_2B_R have been provided. The downstream signaling of GPCR activated by ligand are canonical G protein pathway and non-canonical β-arrestins pathway [55]. In this study, we found that Gq protein inhibitor or knockout of β-arrestin1 failed to affect, while ablation of β-arrestin2 reversed the inhibitory functions of fluoxetine in A1 astrocyte reactivity in vitro. Results in vivo shown that compared with WT mice, fluoxetine administration did not affect abnormal behaviors and activation of A1 astrocyte in β-arrestin2 knockout mice after CMS stimulation. These data suggested that neither G protein nor β-arrestin1 pathway participated in, whereas β-arrestin2 pathway contributed to the therapeutic actions of fluoxetine, indicating that the anti-depressive function of fluoxetine is mediated by inhibiting A1 astrocyte activation through astrocytic 5-HT_2B_R/β-arrestin2 signaling. As Gq activation is involved in 5-HT_2B_R-mediated cardiac hypertrophy that is a side-effect of 5-HT_2B_R agonist [53], β-arrestin2-biased 5-HT_2B_R ligands would be a promising strategy for the development of a novel antidepressant.

## Conclusions

**Fig.8:**
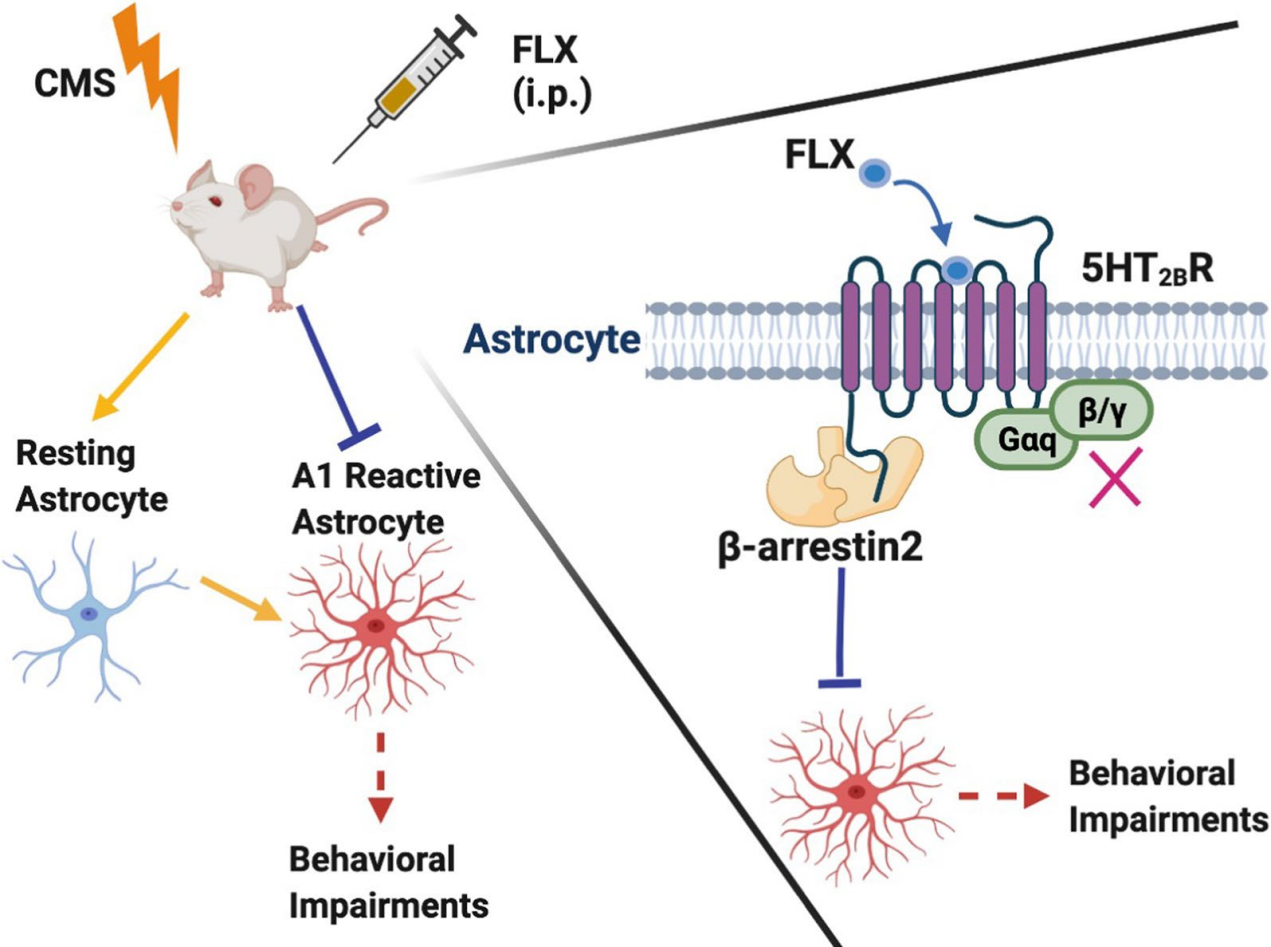
Schematic diagram showing inhibitory effects of fluoxetine on of A1 astrocyte reactivity in depression. Fluoxetine (FLX) administration inhibits the activation of A1 astrocytes in CMS mice and primary astrocytes via 5-HT_2B_R/β-arrestin2 pathway not Gq protein pathway in astrocytes, which could improve behavioral impairments of CMS mice

In summary, this work indicates that fluoxetine administration improves behavioral impairments of CMS mice and inhibits the activation of A1 astrocyte in MDD in vivo and in vitro. These functions of fluoxetine are mediated by astrocytic 5-HT_2B_R through β-arrestin2 signaling and independent of either Gq protein or β-arrestin1 signaling (Fig. 8). Collectively, these results extend our understanding on β-arrestin2-biased astrocytic 5-HT_2B_R signaling and shed light on potential new therapeutic strategy for MDD.

## Supplementary Information

**Additional file 1: Table S1. **Primers and siRNA used in the study. **Fig. S1. **CMS-induced anxiety-like behaviors are reduced by fluoxetine administration. **A** Schematic diagram of CMS preferment and fluoxetine treatment experimental design. **B-D** The circuit diagram (**B**), bouts of mice in center (**C)** and duration of mice staying in center (**D**) in the OFT. **E** Latency time of mice to the first sniff in SIT. **F **Latency time of mice to the first feed > 3 s in NSFT. Quantitative data are mean ± s.e. Data were analyzed using two-way ANOVA, then combined with Tukey’s multiple comparison to assess the differences between groups. n = 8 per group, biologically independent animals. *P < 0.05, **P < 0.01, and ***P < 0.001. **Fig. S2. A**1 astrocyte reactivity in cortex of mice is protected by fluoxetine administration. **A** Heatmap of A1-special and A2-special transcripts in the cortex as analyzed by RT-qPCR. ***P < 0.001 vs control mice treated with saline; ^*###*^*P* < 0.001 vs CMS mice treated with saline. **B-C** Expression of C3 in the cortex. **D** Protein levels of IL-6 in the serum, cortex and hippocampus. Quantitative data are mean ± s.e. Data were analyzed using two-way ANOVA, then combined with Tukey’s multiple comparison to assess the differences between groups. *n* = 4 per group, biologically independent animals. *P < 0.05, **P < 0.01, and ***P < 0.001. **Fig. S3. **Effects of fluoxetine on the releases of LDH in the supernatants of astrocyte. **A** Primary cultured microglia, astrocyte and neuron were sained with Iba 1, GFAP and Map2 (Scale bars, 50 µm), respectively. **B**–**D** Effects of fluoxetine on the releases of LDH in the supernatants of astrocyte after A1 (**B**), activated (LPS)-MCM (**C**) and IL-6 (**D**) stimulation. Quantitative data are mean ± s.e. Data were analyzed using two-way ANOVA, then combined with Tukey’s multiple comparison to assess the differences between groups. All data represent the results of three independent experiments. *P < 0.05, **P < 0.01, and ***P < 0.001. **Fig. S4 **Expression of 5-HT_2B_R in astrocyte after transfection with siRNA for 48h. **A **Representative blots. **B** Quantitative data shown in (**A**). Quantitative data are mean ± s.e. Data were analyzed using Student’s t test. All data represent the results of three independent experiments. ***P < 0.001. **Fig. S5** AAV-mediated depletion of 5-HT_2B_R in astrocyte. **A **Schematic representation of the AAV virus construct used to express 5-HT_2B_R or control siRNA in astrocyte under the gfaABC1D promoter. **B **Protein levels of 5-HT_2B_R in the hippocampus of mice after AAV injection for 4 weeks. Quantitative data are mean ± s.e. Data were analyzed using Student’s t test. All data represent the results of three independent experiments. ***P < 0.001. **C** Expression of 5-HT_2B_R on astrocyte in the hippocampus DG of mice. Scale bars, 20 µm. Similar results were obtained in three separate experiments. **D** Schematic diagram of AAV injection experimental design. **Fig. S6. **Astrocytic 5-HT_2B_R knockdown reverses the functions of fluoxetine in anxiety-like behaviors and the expression of A1-special markers in cortex of mice. **A**–**C** The circuit diagram (**A**), bouts of mice in center (**B**) and duration of mice with or without fluoxetine treatment after AAV injection staying in center (**C**) in the OFT. **D** Latency time of mice to the first sniff in SIT. **E** Latency time of mice to the first feed > 3 s in NSFT. **F** Levels of A1-special genes in the cortex of CON and CMS mice after AAV injection. Quantitative data are mean ± s.e. Data were analyzed using two-way ANOVA, then combined with Tukey’s multiple comparison to assess the differences between groups. **B-E**: n = 8 (**C**,** E**), *n* = 9 (**B**, **D**) CON with Control AAV and saline; *n* = 8 (**C**,** E**), *n* = 9 (**B**, **D**) CON with Control AAV and FLX; n = 8 (**D**, **E**), *n* = 9 (**B**, **C**) CMS with Control AAV and saline; *n* = 8 (**B**, **E**), *n* = 9 (**C, D**) CMS with Control AAV and FLX; *n* = 8 CON with mHT_2B_R AAV and saline; *n* = 8 (**D**,** E**), *n* = 9 (**B**, **C**) CON with mHT_2B_R AAV and FLX; n = 8 (**E**), *n* = 9 (**B**–**D**) CMS with mHT_2B_R AAV and saline; *n* = 8 (**B, D, E**), *n* = 9 (**C**) CMS with mHT_2B_R AAV and FLX; **F**: *n* = 4 per group; biologically independent animals. *P < 0.05, and ***P < 0.001 vs respective control mice treated with saline; ^*#*^*P* < 0.05, and ^*###*^*P* < 0.001 vs CMS mice injected with control AAV and treated with saline; ^*$*^*P* < 0.05, ^*$$*^*P* < 0.01, and ^*$$$*^*P* < 0.001. **Fig. S7. **β-arrestin 2 is contribute to the effects of fluoxetine on A1 astrocyte reactivity in vitro*.* **A** Expression of β-arrestin 1 in primary astrocyte isolated from WT and β-arrestin 1 knockout mice. **B** Expression of β-arrestin 2 in primary astrocyte of isolated from WT and β-arrestin 2 knockout mice. **C** Effects of β-arrestin 2 knockout on the levels of A1-special genes in astrocyte with or without fluoxetine treatment after activated (LPS)-MCM stimulation. Quantitative data are mean ± s.e. Data were analyzed using two-way ANOVA, then combined with Tukey’s multiple comparison to assess the differences between groups. All data represent the results of three independent experiments. ***P < 0.001 vs respective control cells treated with non-activated MCM; ^*##*^*P* < 0.01, and ^*###*^*P* < 0.001 vs WT control cells treated with LPS-MCM stimulation; ^*$$*^*P* < 0.01, and ^*$$$*^*P* < 0.001. **Fig. S8. **β-arrestin 2 knockout reduces the effects of fluoxetine on the expression of A1-special markers in cortex of CMS mice. **A-C** The circuit diagram (**A**), bouts of WT and β-arrestin 2 knockout mice in center (**B**) and duration of WT and β-arrestin 2 knockout mice staying in center (**C**) in the OFT. **D** Latency time of mice to the first sniff in SIT. **E** Latency time of mice to the first feed > 3 s in NSFT. **F** Levels of A1-special genes in the cortex of WT and β-arrestin 2^−/−^ mice. Data were analyzed using two-way ANOVA, then combined with Tukey’s multiple comparison to assess the differences between groups. Quantitative data are mean ± s.e. **B-E**: *n* = 8 (**D**, **E**), *n* = 9 (**B**,** C**) WT CON with saline; *n* = 8 (**C-E**), *n* = 9 (**B**) WT CON with FLX; n = 8 (**D**, E), *n* = 10 (**B**, **C**) WT CMS with saline; *n* = 8 (**D**, **E**), *n* = 9 (**B**,** C**) WT CMS with FLX; n = 8 (**E**), n = 9 (**B**, **C**), n = 10 (**D**) β-arrestin 2^−/−^ CON with saline; *n* = 8 (**B**, **D**, **E**), *n* = 9 (**C**) β-arrestin 2^−/−^ CON with FLX; n = 8 β-arrestin 2^−/−^ CMS with saline; *n* = 8 (**D**, **E**), *n* = 9 (**B**, **C**) β-arrestin 2^−/−^ CMS with FLX; **F**: *n* = 4 per group; biologically independent animals. ***P < 0.001 vs respective control mice treated with saline; ^#^*P* < 0.05, and ^###^*P* < 0.001 vs WT CMS mice treated with saline; ^$^*P* < 0.05, ^$$^*P* < 0.01, and ^$$$^*P* < 0.001. **Fig. S9.** The overall experimental design of this study.

**Additional file 2. **ANOVA results.

## Data Availability

Supporting data and information about used material are available from the corresponding author on reasonable request.

## Abbreviations

5-HT_2B_R: 5-HT_2B_ receptor
AAV: Adeno-associated virus
CMS: Chronic mild stress
CNS: Central nervous system
FBS: Fetal bovine serum
FLX: Fluoxetine
FST: Forced swimming test
GFAP: Glial fibrillary acidic protein
GPCR: G protein-coupled receptor
Iba1: Ionized calcium binding adapter molecule 1
IF: Immunofluorescence
IHC: Immunohistochemistry
IL: Interleukin
LDH: Lactate dehydrogenase
LPS: Lipopolysaccharide
MAP2: Microtubule-associated protein 2
MCM: Microglia-conditioned medium
MDD: Major depressive disorder
NC: Negative control
NDDs: Neurodegenerative diseases
NSFT: Novelty-suppressed feeding test
OFT: Open field test
PD: Parkinson’s disease
PLL: Poly-L-lysine
RT: Room temperature
RT-qPCR: Quantitative real-time PCR
SERT: Serotonin transporter
siRNA: Small interfering RNA
SIT: Social interaction test
SPT: Sucrose preference test
SSRIs: Selective serotonin receptor inhibitors
TNF: Tumor necrosis factor
TST: Tail suspension test
