# Supplementary material for: Fluoxetine inhibited the activation of A1 reactive astrocyte in a mouse model of major depressive disorder through astrocytic 5-HT2BR/β-arrestin2 pathway
Source: J Neuroinflammation. 2022 Jan 29;19:23. doi: 10.1186/s12974-022-02389-y (PMC8800238; doi:10.1186/s12974-022-02389-y)
Supplement: Supplementary file 1 — Additional file 1: Table S1. Primers and siRNA used in the study. Fig. S1. CMS-induced anxiety-like behaviors are reduced by fluoxetine administration. A Schematic diagram of CMS preferment and fluoxetine treatment experimental design. B-D The circuit diagram (B), bouts of mice in center (C) and duration of mice staying in center (D) in the OFT. E Latency time of mice to the first sniff in SIT. F Latency time of mice to the first feed > 3 s in NSFT. Quantitative data are mean ± s.e. Data were analyzed using two-way ANOVA, then combined with Tukey’s multiple comparison to assess the differences between groups. n = 8 per group, biologically independent animals. *P < 0.05, **P < 0.01, and ***P < 0.001. Fig. S2. A1 astrocyte reactivity in cortex of mice is protected by fluoxetine administration. A Heatmap of A1-special and A2-special transcripts in the cortex as analyzed by RT-qPCR. ***P < 0.001 vs control mice treated with saline; ###P < 0.001 vs CMS mice treated with saline. B-C Expression of C3 in the cortex. D Protein levels of IL-6 in the serum, cortex and hippocampus. Quantitative data are mean ± s.e. Data were analyzed using two-way ANOVA, then combined with Tukey’s multiple comparison to assess the differences between groups. n = 4 per group, biologically independent animals. *P < 0.05, **P < 0.01, and ***P < 0.001. Fig. S3. Effects of fluoxetine on the releases of LDH in the supernatants of astrocyte. A Primary cultured microglia, astrocyte and neuron were sained with Iba 1, GFAP and Map2 (Scale bars, 50 µm), respectively. B–D Effects of fluoxetine on the releases of LDH in the supernatants of astrocyte after A1 (B), activated (LPS)-MCM (C) and IL-6 (D) stimulation. Quantitative data are mean ± s.e. Data were analyzed using two-way ANOVA, then combined with Tukey’s multiple comparison to assess the differences between groups. All data represent the results of three independent experiments. *P < 0.05, **P < 0.01, and ***P < 0.001. Fig. S4 Expression of 5-HT2BR i [file 12974_2022_2389_MOESM1_ESM.docx]

**Additional file 1: Table S1 Primers and siRNA used in the study.**

| Primer | Forward sequence | Reverse sequence |
| --- | --- | --- |
| H2-T23 | GGACCGCGAATGACATAGC | GCACCTCAGGGTGACTTCAT |
| Serping1 | ACAGCCCCCTCTGAATTCTT | GGATGCTCTCCAAGTTGCTC |
| H2-D1 | TCCGAGATTGTAAAGCGTGAAGA | ACAGGGCAGTGCAGGGATAG |
| Ggta1 | GTGAACAGCATGAGGGGTTT | GTTTTGTTGCCTCTGGGTGT |
| Ligp1 | GGGGCAATAGCTCATTGGTA | ACCTCGAAGACATCCCCTTT |
| Gbp2 | GGGGTCACTGTCTGACCACT | GGGAAACCTGGGATGAGATT |
| Fbln5 | CTTCAGATGCAAGCAACAA | CCTATGGGTCACTTGCCACT |
| Ugt1a | CCTATGGGTCACTTGCCACT | AAAACCATGTTGGGCATGAT |
| Fkbp5 | TATGCTTATGGCTCGGCTGG | CAGCCTTCCAGGTGGACTTT |
| Psmb8 | CAGTCCTGAAGAGGCCTACG | CACTTTCACCCAACCGTCTT |
| Srgn | GCAAGGTTATCCTGCTCGGA | TGGGAGGGCCGATGTTATTG |
| Amigo2 | GAGGCGACCATAATGTCGTT | GCATCCAACAGTCCGATTCT |
| C3 | AAAAGGGGCGCAACAAGTTC | GATGCCTTCCGGGTTCTCAA |
| Clcf1 | CTTCAATCCTCCTCGACTGG | TACGTCGGAGTTCAGCTGTG |
| Ptx3 | AACAAGCTCTGTTGCCCATT | TCCCAAATGGAACATTGGAT |
| S100a10 | CCTCTGGCTGTGGACAAAAT | CTGCTCACAAGAAGCAGTGG |
| Sphk1 | GATGCATGAGGTGGTGAATG | TGCTCGTACCCAGCATAGTG |
| Cd109 | CACAGTCGGGAGCCCTAAAG | GCAGCGATTTCGATGTCCAC |
| Ptgs2 | GCTGTACAAGCAGTGGCAAA | CCCCAAAGATAGCATCTGGA |
| Emp1 | GAGACACTGGCCAGAAAAGC | GCAGCGATTTCGATGTCCAC |
| Slc10a6 | GCTTCGGTGGTATGATGCTT | CCACAGGCTTTTCTGGTGAT |
| Tm4sf1 | GCCCAAGCATATTGTGGAGT | AGGGTAGGATGTGGCACAAG |
| B3gnt5 | CGTGGGGCAATGAGAACTAT | CCCAGCTGAACTGAAGAAGG |
| Cd14 | GGACTGATCTCAGCCCTCTG | GCTTCAGCCCAGTGAAAGAC |
| GAPDH | CCTGGAGAAACCTGCCAAGTA | TCATACCAGGAAATGAGCTTGAC |
| 5-HT_2B_R siRNA1 | GGGAAGCAUUUGGCAGGUAUU | UACCUGCCAAAUGCUUCCCUUU |
| 5-HT_2B_R siRNA2 | CCAUCCCAGUCCCUAUUAATT | UUAAUAGGGACUGGGAUGGTT |

**Additional file 1: Fig. S1 CMS-induced anxiety-like behaviors are reduced by fluoxetine administration.** **A** Schematic diagram of CMS preferment and fluoxetine treatment experimental design. **B-D** The circuit diagram (**B**), bouts of mice in center (**C**) and duration of mice staying in center (**D**) in the OFT. **E** The latency time of mice to the first sniff in SIT. **F** The latency time of mice to the first feed > 3 s in NSFT. Quantitative data are mean ± s.e. Data were analyzed using two-way ANOVA, then combined with Tukey’s multiple comparison to assess the differences between groups. n = 8 per group, biologically independent animals. **P* < 0.05, ***P* < 0.01, and ****P* < 0.001.

**Additional file 1: Fig. S2 A1 astrocyte reactivity in cortex of mice is protected by fluoxetine administration.** **A** Heatmap of A1-special and A2-special transcripts in the cortex as analyzed by RT-qPCR. ****P* < 0.001 vs control mice treated with saline; *^###^P* < 0.001 vs CMS mice treated with saline. **B-C** The expression of C3 in the cortex. **D** The protein levels of IL-6 in the serum, cortex and hippocampus. Quantitative data are mean ± s.e. Data were analyzed using two-way ANOVA, then combined with Tukey’s multiple comparison to assess the differences between groups. n = 4 per group, biologically independent animals. **P* < 0.05, ***P* < 0.01, and ****P* < 0.001.

**Additional file 1: Fig. S3 The effects of fluoxetine on the releases of LDH in the supernatants of astrocyte. A** Primary cultured microglia, astrocyte and neuron were sained with Iba 1, GFAP and Map2 (Scale bars, 50 µm), respectively. **B-D** The effects of fluoxetine on the releases of LDH in the supernatants of astrocyte after A1 (**B**), activated (LPS)-MCM (**C**) and IL-6 (**D**) stimulation. Quantitative data are mean ± s.e. Data were analyzed using two-way ANOVA, then combined with Tukey’s multiple comparison to assess the differences between groups. All data represent the results of three independent experiments. **P* < 0.05, ***P* < 0.01, and ****P* < 0.001.

**Additional file 1: Fig. S4 The expression of 5-HT_2B_R in astrocyte after transfection with siRNA for 48h**. **A** Representative blots. **B** Quantitative data shown in (A). Quantitative data are mean ± s.e. Data were analyzed using Student’s t test. All data represent the results of three independent experiments. ****P* < 0.001.

**Additional file 1: Fig. S5 AAV-mediated depletion of 5-HT_2B_R in astrocyte.** **A** Schematic representation of the AAV virus construct used to express 5-HT_2B_R or control siRNA in astrocyte under the gfaABC1D promoter. **B** The protein levels of 5-HT_2B_R in the hippocampus of mice after AAV injection for 4 weeks. Quantitative data are mean ± s.e. Data were analyzed using Student’s t test. All data represent the results of three independent experiments. ****P* < 0.001. **C** The expression of 5-HT_2B_R on astrocyte in the hippocampus DG of mice. Scale bars, 20 µm. Similar results were obtained in three separate experiments. **D** Schematic diagram of AAV injection experimental design.

**Additional file 1: Fig. S6 Astrocytic 5-HT_2B_R knockdown reverses the functions of fluoxetine in anxiety-like behaviors and the expression of A1-special markers in cortex of mice.** **A-C** The circuit diagram (**A**), bouts of mice in center (**B**) and duration of mice with or without fluoxetine treatment after AAV injection staying in center (**C**) in the OFT. **D** The latency time of mice to the first sniff in SIT. **E** The latency time of mice to the first feed > 3 s in NSFT. **F** The levels of A1-special genes in the cortex of CON and CMS mice after AAV injection. Quantitative data are mean ± s.e. Data were analyzed using two-way ANOVA, then combined with Tukey’s multiple comparison to assess the differences between groups. **B-E**: n = 8 (**C**, **E**), n = 9 (**B**, **D**) CON with Control AAV and saline; n = 8 (**C**, **E**), n = 9 (**B**, **D**) CON with Control AAV and FLX; n = 8 (**D**, **E**), n = 9 (**B**, **C**) CMS with Control AAV and saline; n = 8 (**B, E**), n = 9 (**C, D**) CMS with Control AAV and FLX; n = 8 CON with mHT_2B_R AAV and saline; n = 8 (**D**, **E**), n = 9 (**B**, **C**) CON with mHT_2B_R AAV and FLX; n = 8 (**E**), n = 9 (**B-D**) CMS with mHT_2B_R AAV and saline; n = 8 (**B, D, E**), n = 9 (**C**) CMS with mHT_2B_R AAV and FLX; **F**: n = 4 per group; biologically independent animals. **P* < 0.05, and ****P* < 0.001 vs respective control mice treated with saline; *^#^P* < 0.05, and *^###^P* < 0.001 vs CMS mice injected with control AAV and treated with saline; *^$^P* < 0.05, *^$$^P* < 0.01, and *^$$$^P* < 0.001.

**

**Additional file 1: Fig. S7 β-arrestin 2 is contribute to the effects of fluoxetine on A1 astrocyte reactivity *in vitro*.** **A** The expression of β-arrestin 1 in primary astrocyte isolated from WT and β-arrestin 1 knockout mice. **B** The expression of β-arrestin 2 in primary astrocyte of isolated from WT and β-arrestin 2 knockout mice. **C** Effects of β-arrestin 2 knockout on the levels of A1-special genes in astrocyte with or without fluoxetine treatment after activated (LPS)-MCM stimulation. Quantitative data are mean ± s.e. Data were analyzed using two-way ANOVA, then combined with Tukey’s multiple comparison to assess the differences between groups. All data represent the results of three independent experiments. ****P* < 0.001 vs respective control cells treated with non-activated MCM; *^##^P* < 0.01, and *^###^P* < 0.001 vs WT control cells treated with LPS-MCM stimulation; *^$$^P* < 0.01, and *^$$$^P* < 0.001.

**Additional file 1: Fig. S8 β-arrestin 2 knockout reduces the effects of fluoxetine on the expression of A1-special markers in cortex of CMS mice.** **A-C** The circuit diagram (**A**), bouts of WT and β-arrestin 2 knockout mice in center (**B**) and duration of WT and β-arrestin 2 knockout mice staying in center (**C**) in the OFT. **D** The latency time of mice to the first sniff in SIT. **E** The latency time of mice to the first feed > 3 s in NSFT. **F** The levels of A1-special genes in the cortex of WT and β-arrestin 2^-/-^ mice. Data were analyzed using two-way ANOVA, then combined with Tukey’s multiple comparison to assess the differences between groups. Quantitative data are mean ± s.e. **B-E**: n = 8 (**D**, **E**), n = 9 (**B**, **C**) WT CON with saline; n = 8 (**C-E**), n = 9 (**B**) WT CON with FLX; n = 8 (**D**, **E**), n = 10 (**B**, **C**) WT CMS with saline; n = 8 (**D**, **E**), n = 9 (**B**, **C**) WT CMS with FLX; n = 8 (**E**), n = 9 (**B**, **C**), n = 10 (**D**) β-arrestin 2^-/-^ CON with saline; n = 8 (**B**, **D**, **E**), n = 9 (**C**) β-arrestin 2^-/-^ CON with FLX; n = 8 β-arrestin 2^-/-^ CMS with saline; n = 8 (**D**, **E**), n = 9 (**B**, **C**) β-arrestin 2^-/-^ CMS with FLX; **F**: n = 4 per group; biologically independent animals. ****P* < 0.001 vs respective control mice treated with saline; *^#^P* < 0.05, and *^###^P* < 0.001 vs WT CMS mice treated with saline; *^$^P* < 0.05, *^$$^P* < 0.01, and *^$$$^P* < 0.001.

**Additional file 1: Fig. S9 The overall experimental design of this study.**
