# Supplementary material for: Fluoxetine inhibited the activation of A1 reactive astrocyte in a mouse model of major depressive disorder through astrocytic 5-HT2BR/β-arrestin2 pathway
Source: J Neuroinflammation. 2022 Jan 29;19:23. doi: 10.1186/s12974-022-02389-y (PMC8800238; doi:10.1186/s12974-022-02389-y)
Supplement: Supplementary file 2 — Additional file 2. ANOVA results. [file 12974_2022_2389_MOESM2_ESM.docx]

**ANOVA results**

**Fig. 1**

**A**: Interaction F (30, 304) = 4.431, *P <* 0.0001; time effect F (10, 304) = 7.035, *P <* 0.0001; stimulation and treatment effect F (3, 304) = 74.05, *P <* 0.0001;

**B**: Interaction F (1, 28) = 6.628, *P* = 0.0156; treatment effect F (1, 28) = 5.226, *P* = 0.03; stimulation effect F (1, 28) = 11.85, *P =* 0.0018;

**C**: Interaction F (1, 30) = 2.365, *P* = 0.1346; treatment effect F (1, 30) = 9.116, *P* = 0.0051; stimulation effect F (1, 30) = 7.398, *P* = 0.0108;

**E**: Interaction F (1, 12) = 51.65, *P <* 0.0001; treatment effect F (1, 12) = 41.52, *P <* 0.0001; stimulation effect F (1, 12) = 93.49, *P <* 0.0001;

**G**: Interaction F (1, 12) = 11.88, *P* = 0.0048; treatment effect F (1, 12) = 0.6922, *P* = 0.4217; stimulation effect F (1, 12) = 30.31, *P* = 0.0001;

**H**: H2-T23: Interaction F (1, 12) = 0.8006, *P* = 0.3885; treatment effect F (1, 12) = 2.227, *P* = 0.1614; stimulation effect F (1, 12) = 0.04677, *P* = 0.8324;

Serping1: Interaction F (1, 12) = 175.2, *P <* 0.0001; treatment effect F (1, 12) = 61.9, *P <* 0.0001; stimulation effect F (1, 12) = 79.99, *P <* 0.0001;

H2-D1: Interaction F (1, 12) = 0.2595, *P* = 0.6197; treatment effect F (1, 12) = 11.95, *P* = 0.0047; stimulation effect F (1, 12) = 1.821, *P* = 0.2022;

Ggta1: Interaction F (1, 12) = 0.06882, *P* = 0.7975; treatment effect F (1, 12) = 1.356, *P* = 0.2668; stimulation effect F (1, 12) = 1.293, *P* = 0.2776;

Ligp1: Interaction F (1, 12) = 729.4, *P <* 0.0001; treatment effect F (1, 12) = 517, *P <* 0.0001; stimulation effect F (1, 12) = 487.9, *P <* 0.0001;

Gbp2: Interaction F (1, 12) = 0.06433, *P* = 0.8041; treatment effect F (1, 12) = 0.03254, *P* = 0.8599; stimulation effect F (1, 12) = 0.008722, *P* = 0.9271;

Fbln5: Interaction F (1, 12) = 0.2137, *P* = 0.6521; treatment effect F (1, 12) = 0.2728, *P* = 0.6109; stimulation effect F (1, 12) = 0.1618, *P* = 0.6945;

Ugt1a: Interaction F (1, 12) = 0.007855, *P* = 0.9308; treatment effect F (1, 12) = 0.6252, *P* = 0.4445; stimulation effect F (1, 12) = 2.659, *P* = 0.1289;

Fkbp5: Interaction F (1, 12) = 0.1164, *P* = 0.7389; treatment effect F (1, 12) = 0.5304, *P* = 0.4804; stimulation effect F (1, 12) = 1.59, *P* = 0.2313;

Psmb8: Interaction F (1, 12) = 96.54, *P <* 0.0001; treatment effect F (1, 12) = 54.19, *P <* 0.0001; stimulation effect F (1, 12) = 45.22, *P <* 0.0001;

Srgn: Interaction F (1, 12) = 0.2015, *P* = 0.6615; treatment effect F (1, 12) = 0.3744, *P* = 0.552; stimulation effect F (1, 12) = 0.3348, *P* = 0.5735;

Amigo2: Interaction F (1, 12) = 101, *P <* 0.0001; treatment effect F (1, 12) = 36.83, *P <* 0.0001; stimulation effect F (1, 12) = 34, *P <* 0.0001;

C3: Interaction F (1, 12) = 4.748, *P* = 0.05; treatment effect F (1, 12) = 9.003, *P* = 0.0111; stimulation effect F (1, 12) = 16.54, *P* = 0.0016;

Clcf1: Interaction F (1, 12) = 0.6293, *P* = 0.443; treatment effect F (1, 12) = 8.579, *P* = 0.0126; stimulation effect F (1, 12) = 0.4917, *P* = 0.4965;

Ptx3: Interaction F (1, 12) = 0.004044, *P* = 0.9503; treatment effect F (1, 12) = 0.1941, *P* = 0.6674; stimulation effect F (1, 12) = 3.469, *P* = 0.0872;

S100a10: Interaction F (1, 12) = 1.389, *P* = 0.2614; treatment effect F (1, 12) = 3.118, *P* = 0.1028; stimulation effect F (1, 12) = 0.1511, *P* = 0.7043;

Sphk1: Interaction F (1, 12) = 7.67, *P* = 0.017; treatment effect F (1, 12) = 0.1671, *P* = 0.6899; stimulation effect F (1, 12) = 0.6895, *P* = 0.4226;

Cd109: Interaction F (1, 12) = 4.885, *P* = 0.0473; treatment effect F (1, 12) = 1.025, *P* = 0.3314; stimulation effect F (1, 12) = 0.0222, *P* = 0.884;

Ptgs2: Interaction F (1, 12) = 4.387, *P* = 0.0581; treatment effect F (1, 12) = 5.915, *P* = 0.0316; stimulation effect F (1, 12) = 0.1123, *P* = 0.7433;

Emp1: Interaction F (1, 12) = 0.7338, *P* = 0.4084; treatment effect F (1, 12) = 0.2959, *P* = 0.5964; stimulation effect F (1, 12) = 2.609, *P* = 0.1322;

Slc10a6: Interaction F (1, 12) = 0.007966, *P* = 0.9304; treatment effect F (1, 12) = 8.299, *P* = 0.0138; stimulation effect F (1, 12) = 2.104, *P* = 0.1726;

Tm4sf1: Interaction F (1, 12) = 2.123, *P* = 0.1708; treatment effect F (1, 12) = 3.632, *P* = 0.0809; stimulation effect F (1, 12) = 11.69, *P* = 0.0051;

B3gnt5: Interaction F (1, 12) = 5.258, *P* = 0.0407; treatment effect F (1, 12) = 0.9746, *P* = 0.343; stimulation effect F (1, 12) = 0.322, *P* = 0.5809;

Cd14: Interaction F (1, 12) = 0.1325, *P* = 0.7222; treatment effect F (1, 12) = 3.036, *P* = 0.107; stimulation effect F (1, 12) = 3.874, *P* = 0.0726;

**J**: Interaction F (1, 12) = 53.98, *P <* 0.0001; treatment effect F (1, 12) = 24.89, *P* = 0.0003; stimulation effect F (1, 12) = 132.4, *P <* 0.0001;

**L**: Interaction F (1, 12) = 92.73, *P <* 0.0001; treatment effect F (1, 12) = 92.41, *P <* 0.0001; stimulation effect F (1, 12) = 144.2, *P <* 0.0001;

**N**: Interaction F (1, 12) = 125.8, *P <* 0.0001; treatment effect F (1, 12) = 110.2, *P <* 0.0001; stimulation effect F (1, 12) = 283.7, *P <* 0.0001.

**Fig. 2**

**A**: H2-T23: Interaction F (1, 8) = 74.93, *P <* 0.0001; treatment effect F (1, 8) = 42.81, *P* = 0.0002; stimulation effect F (1, 8) = 945.7, *P <* 0.0001;

Serping1: Interaction F (1, 8) = 144.8, *P <* 0.0001; treatment effect F (1, 8) = 56.02, *P <* 0.0001; stimulation effect F (1, 8) = 234.9, *P <* 0.0001;

H2-D1: Interaction F (1, 8) = 29.12, *P* = 0.0006; treatment effect F (1, 8) = 12.02, *P* = 0.0085; stimulation effect F (1, 8) = 696.8, *P <* 0.0001;

Ggta1: Interaction F (1, 8) = 371.1, *P <* 0.0001; treatment effect F (1, 8) = 4.208, *P* = 0.0743; stimulation effect F (1, 8) = 58.91, *P <* 0.0001;

Ligp1: Interaction F (1, 8) = 563.2, *P <* 0.0001; treatment effect F (1, 8) = 438.4, *P <* 0.0001; stimulation effect F (1, 8) = 3243, *P <* 0.0001;

Gbp2: Interaction F (1, 8) = 123.9, *P <* 0.0001; treatment effect F (1, 8) = 253.9, *P <* 0.0001; stimulation effect F (1, 8) = 4923, *P <* 0.0001;

Fbln5: Interaction F (1, 8) = 133, *P <* 0.0001; treatment effect F (1, 8) = 1053, *P <* 0.0001; stimulation effect F (1, 8) = 2301, *P <* 0.0001;

Ugt1a: Interaction F (1, 8) = 164.9, *P <* 0.0001; treatment effect F (1, 8) = 348.6, *P <* 0.0001; stimulation effect F (1, 8) = 439.5, *P <* 0.0001;

Fkbp5: Interaction F (1, 8) = 12.29, *P* = 0.008; treatment effect F (1, 8) = 5.087, *P* = 0.0541; stimulation effect F (1, 8) = 4.456, *P* = 0.0678;

Psmb8: Interaction F (1, 8) = 170.3, *P <* 0.0001; treatment effect F (1, 8) = 96.02, *P <* 0.0001; stimulation effect F (1, 8) = 1246, *P <* 0.0001;

Srgn: Interaction F (1, 8) = 378.8, *P <* 0.0001; treatment effect F (1, 8) = 343, *P <* 0.0001; stimulation effect F (1, 8) = 397.2, *P <* 0.0001;

Amigo2: Interaction F (1, 8) = 7.423, *P* = 0.0261; treatment effect F (1, 8) = 12.18, *P* = 0.0082; stimulation effect F (1, 8) = 47.83, *P* = 0.0001;

C3: Interaction F (1, 8) = 12.7, *P* = 0.0074; treatment effect F (1, 8) = 23.11, *P* = 0.0013; stimulation effect F (1, 8) = 2851, *P <* 0.0001;

Clcf1: Interaction F (1, 8) = 0.9144, *P* = 0.367, treatment effect F (1, 8) = 2.358, *P* = 0.1632, stimulation effect F (1, 8) = 6.109, *P* = 0.0386;

Ptx3: Interaction F (1, 8) = 7.973, *P* = 0.0224; treatment effect F (1, 8) = 0.2275, *P* = 0.6462, stimulation effect F (1, 8) = 0.5525, *P* = 0.4786;

S100a10: Interaction F (1, 8) = 0.2698, *P* = 0.6175; treatment effect F (1, 8) = 0.8856, *P* = 0.3742; stimulation effect F (1, 8) = 3.101, *P* = 0.1163;

Sphk1: Interaction F (1, 8) = 2.639, *P* = 0.1429; treatment effect F (1, 8) = 3.541, *P* = 0.0966; stimulation effect F (1, 8) = 0.005966, *P* = 0.9403;

Cd109: Interaction F (1, 8) = 1.921, *P* = 0.2032; treatment effect F (1, 8) = 0.4351, *P* = 0.528; stimulation effect F (1, 8) = 0.06966, *P* = 0.7985;

Ptgs2: Interaction F (1, 8) = 1.801, *P* = 0.2164; treatment effect F (1, 8) = 5.472, *P* = 0.0475; stimulation effect F (1, 8) = 30.26, *P* = 0.0006;

Emp1: Interaction F (1, 8) = 5.335, *P* = 0.0497; treatment effect F (1, 8) = 6.205, *P* = 0.0375; stimulation effect F (1, 8) = 16.4, *P* = 0.0037;

Slc10a6: Interaction F (1, 8) = 0.224, *P* = 0.6486; treatment effect F (1, 8) = 2.113, *P* = 0.1841; stimulation effect F (1, 8) = 0.03343, *P* = 0.8595;

Tm4sf1: Interaction F (1, 8) = 14.03, *P* = 0.0057; treatment effect F (1, 8) = 5.494, *P* = 0.0471; stimulation effect F (1, 8) = 3.331, *P* = 0.1054;

B3gnt5: Interaction F (1, 8) = 19.52, *P* = 0.0022; treatment effect F (1, 8) = 2.338, *P* = 0.1648; stimulation effect F (1, 8) = 0.5259, *P* = 0.489;

Cd14: Interaction F (1, 8) = 12.24, *P* = 0.0081; treatment effect F (1, 8) = 19.76, *P* = 0.0022; stimulation effect F (1, 8) = 106.3, *P <* 0.0001;

**B**: H2-T23: Interaction F (1, 8) = 390.2, *P <* 0.0001 treatment effect F (1, 8) = 19.1, *P* = 0.0024; stimulation effect F (1, 8) = 62733, *P <* 0.0001;

Serping1: Interaction F (1, 8) = 12.61, *P* = 0.0075; treatment effect F (1, 8) = 7.207, *P* = 0.0277; stimulation effect F (1, 8) = 4376, *P <* 0.0001;

H2-D1: Interaction F (1, 8) = 10.18, *P* = 0.0128; treatment effect F (1, 8) = 16.19, *P* = 0.0038; stimulation effect F (1, 8) = 639.5, *P <* 0.0001;

Ggta1: Interaction F (1, 8) = 58.29, *P <* 0.0001; treatment effect F (1, 8) = 44.25, *P* = 0.0002; stimulation effect F (1, 8) = 7.69, *P* = 0.0242;

Ligp1: Interaction F (1, 8) = 21.69, *P* = 0.0016; treatment effect F (1, 8) = 16.43, *P* = 0.0037; stimulation effect F (1, 8) = 5972, *P <* 0.0001;

Gbp2: Interaction F (1, 8) = 8.897, *P* = 0.0175; treatment effect F (1, 8) = 12.98, *P* = 0.007; stimulation effect F (1, 8) = 54.6, *P <* 0.0001;

Fbln5: Interaction F (1, 8) = 8.66, *P* = 0.0186; treatment effect F (1, 8) = 15.88, *P* = 0.004; stimulation effect F (1, 8) = 32.81, *P* = 0.0004;

Ugt1a: Interaction F (1, 8) = 3.095, *P* = 0.1166; treatment effect F (1, 8) = 0.1791, *P* = 0.6833; stimulation effect F (1, 8) = 20.07, *P* = 0.0021;

Fkbp5: Interaction F (1, 8) = 49.37, *P* = 0.0001; treatment effect F (1, 8) = 15.62, *P* = 0.0042; stimulation effect F (1, 8) = 189.2, *P <* 0.0001;

Psmb8: Interaction F (1, 8) = 45.54, *P* = 0.0001; treatment effect F (1, 8) = 14.77, *P* = 0.0049; stimulation effect F (1, 8) = 4085, *P <* 0.0001;

Srgn: Interaction F (1, 8) = 125.8, *P <* 0.0001; treatment effect F (1, 8) = 78.6, *P <* 0.0001; stimulation effect F (1, 8) = 139.5, *P <* 0.0001;

Amigo2: Interaction F (1, 8) = 60.54, *P <* 0.0001; treatment effect F (1, 8) = 56.39, *P <* 0.0001; stimulation effect F (1, 8) = 38.46, *P* = 0.0003;

C3: Interaction F (1, 8) = 32.99, *P* = 0.0004; treatment effect F (1, 8) = 27.01, *P* = 0.0008; stimulation effect F (1, 8) = 355, *P <* 0.0001;

Clcf1: Interaction F (1, 8) = 0.0379, *P* = 0.8505; treatment effect F (1, 8) = 4.544, *P* = 0.0656; stimulation effect F (1, 8) = 5.807, *P* = 0.0425;

Ptx3: Interaction F (1, 8) = 1.86, *P* = 0.2098; treatment effect F (1, 8) = 2.002, *P* = 0.1949; stimulation effect F (1, 8) = 6.755, *P* = 0.0317;

S100a10: Interaction F (1, 8) = 30.53, *P* = 0.0006; treatment effect F (1, 8) = 4.568, *P* = 0.0651; stimulation effect F (1, 8) = 175.8, *P <* 0.0001;

Sphk1: Interaction F (1, 8) = 2.718, *P* = 0.1378; treatment effect F (1, 8) = 11.28, *P* = 0.0099; stimulation effect F (1, 8) = 5.251, *P* = 0.0512;

Cd109: Interaction F (1, 8) = 70.35, *P <* 0.0001; treatment effect F (1, 8) = 76.98, *P <* 0.0001; stimulation effect F (1, 8) = 95.8, *P <* 0.0001;

Ptgs2: Interaction F (1, 8) = 0.4618, *P* = 0.516; treatment effect F (1, 8) = 17.46, *P* = 0.0031; stimulation effect F (1, 8) = 0.1472, *P* = 0.7113;

Emp1: Interaction F (1, 8) = 18.09, *P* = 0.0028; treatment effect F (1, 8) = 12.93, *P* = 0.007; stimulation effect F (1, 8) = 58.87, *P <* 0.0001;

Slc10a6: Interaction F (1, 8) = 0.6927, *P* = 0.4294; treatment effect F (1, 8) = 0.1105, *P* = 0.7482; stimulation effect F (1, 8) = 0.08335, *P* = 0.7801;

Tm4sf1: Interaction F (1, 8) = 1.612, *P* = 0.2398; treatment effect F (1, 8) = 1.92, *P* = 0.2033; stimulation effect F (1, 8) = 2.423, *P* = 0.1581;

B3gnt5: Interaction F (1, 8) = 4.232, *P* = 0.0737; treatment effect F (1, 8) = 1.353, *P* = 0.2782; stimulation effect F (1, 8) = 20.64, *P* = 0.0019;

Cd14: Interaction F (1, 8) = 3.583, *P* = 0.095; treatment effect F (1, 8) = 0.2297, *P* = 0.6445; stimulation effect F (1, 8) = 0.1617, *P* = 0.6981;

**D**: Interaction F (1, 8) = 7.137, *P* = 0.0283; treatment effect F (1, 8) = 18.51, *P* = 0.0026, stimulation effect F (1, 8) = 3.723, *P* = 0.0898;

**F**: Interaction F (1, 8) = 40.36, *P* = 0.0002, treatment effect F (1, 8) = 54.03, *P <* 0.0001; stimulation effect F (1, 8) = 10.84, *P* = 0.011;

**G**: H2-T23: Interaction F (1, 8) = 14.47, *P* = 0.0052; treatment effect F (1, 8) = 1.674, *P* = 0.2318; stimulation effect F (1, 8) = 12.87, *P* = 0.0071;

Serping1: Interaction F (1, 8) = 38.3, *P* = 0.0003; treatment effect F (1, 8) = 112.2, *P <* 0.0001; stimulation effect F (1, 8) = 300.7, *P <* 0.0001;

H2-D1: Interaction F (1, 8) = 2.898, *P* = 0.1271; treatment effect F (1, 8) = 1.237, *P* = 0.2983; stimulation effect F (1, 8) = 16.89, *P* = 0.0034;

Ggta1: Interaction F (1, 8) = 3.512e-005, *P* = 0.9954; treatment effect F (1, 8) = 0.3441, *P* = 0.5736; stimulation effect F (1, 8) = 9.009, *P* = 0.017;

Ligp1: Interaction F (1, 8) = 22.58, *P* = 0.0014; treatment effect F (1, 8) = 86.82, *P <* 0.0001; stimulation effect F (1, 8) = 273.2, *P <* 0.0001;

Gbp2: Interaction F (1, 8) = 3.4, *P* = 0.1024; treatment effect F (1, 8) = 0.00165, *P* = 0.9686; stimulation effect F (1, 8) = 16.17, *P* = 0.0038;

Fbln5: Interaction F (1, 8) = 22.39, *P* = 0.0015; treatment effect F (1, 8) = 5.195, *P* = 0.0521; stimulation effect F (1, 8) = 42.12, *P* = 0.0002;

Ugt1a: Interaction F (1, 8) = 2.699, *P* = 0.139; treatment effect F (1, 8) = 3.187, *P* = 0.1121; stimulation effect F (1, 8) = 0.02424, *P* = 0.8801;

Fkbp5: Interaction F (1, 8) = 8.931, *P* = 0.0174; treatment effect F (1, 8) = 0.1533, *P* = 0.7057; stimulation effect F (1, 8) = 0.3874, *P* = 0.551;

Psmb8: Interaction F (1, 8) = 35.04, *P* = 0.0004; treatment effect F (1, 8) = 19.92, *P* = 0.0021; stimulation effect F (1, 8) = 4.51, *P* = 0.0665;

Srgn: Interaction F (1, 8) = 1.085, *P* = 0.328; treatment effect F (1, 8) = 13.13, *P* = 0.0067; stimulation effect F (1, 8) = 69.69, *P* < 0.0001;

Amigo2: Interaction F (1, 8) = 15.37, *P* = 0.0044; treatment effect F (1, 8) = 1.54, *P* = 0.2498; stimulation effect F (1, 8) = 8.412, *P* = 0.0199;

C3: Interaction F (1, 8) = 1.692, *P* = 0.2295; treatment effect F (1, 8) = 13.16, *P* = 0.0067; stimulation effect F (1, 8) = 17.44, *P* = 0.0031;

Clcf1: Interaction F (1, 8) = 1.766, *P* = 0.2206; treatment effect F (1, 8) = 0.4814, *P* = 0.5074; stimulation effect F (1, 8) = 5.423, *P* = 0.0482;

Ptx3: Interaction F (1, 8) = 0.5025, *P* = 0.4985; treatment effect F (1, 8) = 0.01941, *P* = 0.8926; stimulation effect F (1, 8) = 4.012, *P* = 0.0801;

S100a10: Interaction F (1, 8) = 1.792, *P* = 0.2174; treatment effect F (1, 8) = 0.7358, *P* = 0.416; stimulation effect F (1, 8) = 0.1035, *P* = 0.7559;

Sphk1: Interaction F (1, 8) = 0.07502, *P* = 0.7911; treatment effect F (1, 8) = 0.0007106, *P* = 0.9794; stimulation effect F (1, 8) = 0.002063, *P* = 0.9649;

Cd109: Interaction F (1, 8) = 7.987, *P* = 0.0223; treatment effect F (1, 8) = 0.6452, *P* = 0.445; stimulation effect F (1, 8) = 0.4135, *P* = 0.5382;

Ptgs2: Interaction F (1, 8) = 0.1974, *P* = 0.6686; treatment effect F (1, 8) = 0.2553, *P* = 0.627; stimulation effect F (1, 8) = 0.164, *P* = 0.6961;

Emp1: Interaction F (1, 8) = 2.219, *P* = 0.1747; treatment effect F (1, 8) = 7.218, *P* = 0.0276; stimulation effect F (1, 8) = 1.699, *P* = 0.2286;

Slc10a6: Interaction F (1, 8) = 0.7848, *P* = 0.4015; treatment effect F (1, 8) = 6.222, *P* = 0.0373; stimulation effect F (1, 8) = 0.2333, *P* = 0.642;

Tm4sf1: Interaction F (1, 8) = 2.228, *P* = 0.1738; treatment effect F (1, 8) = 0.06181, *P* = 0.8099; stimulation effect F (1, 8) = 0.0004699, *P* = 0.9832;

B3gnt5: Interaction F (1, 8) = 0.01242, *P* = 0.914; treatment effect F (1, 8) = 1.79, *P* = 0.2177; stimulation effect F (1, 8) = 4.074, *P* = 0.0782;

Cd14: Interaction F (1, 8) = 0.3418, *P* = 0.5749; treatment effect F (1, 8) = 0.2046, *P* = 0.663; stimulation effect F (1, 8) = 15.34, *P* = 0.0044;

**I**: Interaction F (1, 8) = 26.76, *P* = 0.0009; treatment effect F (1, 8) = 45.39, *P* = 0.0001; stimulation effect F (1, 8) = 20.83, *P* = 0.0018.

**Fig. 3**

**C**: Serping1: Interaction F (2, 12) = 201.8, *P <* 0.0001; inhibitor effect F (1, 12) = 212.6, *P <* 0.0001; stimulation and treatment effect F (2, 12) = 771.9, *P <* 0.0001;

Ligp1: Interaction F (2, 12) = 56.38, *P <* 0.0001; inhibitor effect F (1, 12) = 77.57, *P <* 0.0001; stimulation and treatment effect F (2, 12) = 319, *P <* 0.0001;

Psmb8: Interaction F (2, 12) = 19.03, *P* = 0.0002; inhibitor effect F (1, 12) = 36.43, *P <* 0.0001; stimulation and treatment effect F (2, 12) = 168.8, *P <* 0.0001;

Srgn: Interaction F (2, 12) = 33.3, *P <* 0.0001; inhibitor effect F (1, 12) = 45.13, *P <* 0.0001; stimulation and treatment effect F (2, 12) = 124.9, *P <* 0.0001;

Amigo2: Interaction F (2, 12) = 3.861, *P* = 0.0507; inhibitor effect F (1, 12) = 4.086, *P* = 0.0661; stimulation and treatment effect F (2, 12) = 35.01, *P <* 0.0001;

C3: Interaction F (2, 12) = 88.66, *P <* 0.0001; inhibitor effect F (1, 12) = 80.55, *P <* 0.0001; stimulation and treatment effect F (2, 12) = 759.6, *P <* 0.0001;

**D**: Serping1: Interaction F (2, 12) = 17.64, *P* = 0.0003; inhibitor effect F (1, 12) = 20.81, *P* = 0.0007; stimulation and treatment effect F (2, 12) = 55.42, *P <* 0.0001;

Ligp1: Interaction F (2, 12) = 22.08, *P <* 0.0001; inhibitor effect F (1, 12) = 86.78, *P <* 0.0001; stimulation and treatment effect F (2, 12) = 100.3, *P <* 0.0001;

Psmb8: Interaction F (2, 12) = 12.38, *P* = 0.0012; inhibitor effect F (1, 12) = 37.83, *P <* 0.0001; stimulation and treatment effect F (2, 12) = 65.55, *P <* 0.0001;

Srgn: Interaction F (2, 12) = 11.84, *P* = 0.0014; inhibitor effect F (1, 12) = 16.73, *P* = 0.0015; stimulation and treatment effect F (2, 12) = 23.65, *P <* 0.0001;

Amigo2: Interaction F (2, 12) = 5.264, *P* = 0.0228; inhibitor effect F (1, 12) = 13, *P* = 0.0036; stimulation and treatment effect F (2, 12) = 37.61, *P <* 0.0001;

C3: Interaction F (2, 12) = 68.3, *P <* 0.0001; inhibitor effect F (1, 12) = 188.3, *P <* 0.0001; stimulation and treatment effect F (2, 12) = 126.6, *P <* 0.0001;

**E**: Serping1: Interaction F (3, 16) = 81.66, *P <* 0.0001; stimulation and treatment effect F (3, 16) = 334.5, *P <* 0.0001; siRNA effect F (1, 16) = 90.73, *P <* 0.0001;

Ligp1: Interaction F (3, 16) = 130.3, *P <* 0.0001; stimulation and treatment effect F (3, 16) = 603.1, *P <* 0.0001; siRNA effect F (1, 16) = 142.6, *P <* 0.0001;

Psmb8: Interaction F (3, 16) = 30.08, *P <* 0.0001; stimulation and treatment effect F (3, 16) = 131.4, *P <* 0.0001; siRNA effect F (1, 16) = 36.8, *P <* 0.0001;

Srgn: Interaction F (3, 16) = 40.99, *P <* 0.0001; stimulation and treatment effect F (3, 16) = 217.3, *P <* 0.0001; siRNA effect F (1, 16) = 63, *P <* 0.0001;

Amigo2: Interaction F (3, 16) = 28.63, *P <* 0.0001; stimulation and treatment effect F (3, 16) = 96.33, *P <* 0.0001; siRNA effect F (1, 16) = 40.88, *P <* 0.0001;

C3: Interaction F (3, 16) = 10.29, *P* = 0.0005; stimulation and treatment effect F (3, 16) = 43.92, *P <* 0.0001; siRNA effect F (1, 16) = 12.59, *P* = 0.0027.

**Fig. 4**

**A**: Interaction F (70, 680) = 3.425, *P <* 0.0001; time effect F (10, 680) = 20.68, *P <* 0.0001; stimulation and treatment effect F (7, 680) = 51.24, *P <* 0.0001;

**B-TST**: Interaction F (3, 59) = 3.799, *P* = 0.0147; stimulation and treatment effect F (3, 59) = 18.81, *P <* 0.0001; AAV effect F (1, 59) = 1.007, *P* = 0.3198;

**B-FST**: Interaction F (3, 58) = 3.522, *P* = 0.0204; stimulation and treatment effect F (3, 58) = 18.88, *P <* 0.0001; AAV effect F (1, 58) = 2.508, *P* = 0.1187;

**D**: Interaction F (3, 24) = 16.82, *P <* 0.0001; stimulation and treatment effect F (3, 24) = 52.58, *P <* 0.0001; AAV effect F (1, 24) = 21.76, *P <* 0.0001;

**F**: Interaction F (3, 24) = 4.065, *P* = 0.0181; stimulation and treatment effect F (3, 24) = 14.84, *P <* 0.0001; AAV effect F (1, 24) = 3.022, *P* = 0.0949;

**G**: Serping1: Interaction F (3, 24) = 42.99, *P <* 0.0001; stimulation and treatment effect F (3, 24) = 260.8, *P <* 0.0001; AAV effect F (1, 24) = 57.57, *P <* 0.0001;

Ligp1: Interaction F (3, 24) = 256.4, *P <* 0.0001; stimulation and treatment effect F (3, 24) = 104, *P <* 0.0001; AAV effect F (1, 24) = 295.9, *P <* 0.0001;

Psmb8: Interaction F (3, 24) = 61.68, *P <* 0.0001; stimulation and treatment effect F (3, 24) = 240.6, *P <* 0.0001; AAV effect F (1, 24) = 57.38, *P <* 0.0001;

Amigo2: Interaction F (3, 24) = 16.43, *P <* 0.0001; stimulation and treatment effect F (3, 24) = 79.12, *P <* 0.0001; AAV effect F (1, 24) = 48.37, *P <* 0.0001;

C3: Interaction F (3, 24) = 47.19, *P <* 0.0001; stimulation and treatment effect F (3, 24) = 187.4, *P <* 0.0001; AAV effect F (1, 24) = 33.19, *P <* 0.0001.

**Fig. 5**

**A**: Serping1: Interaction F (2, 12) = 0.8954, *P* = 0.4341; inhibitor effect F (1, 12) = 0.0797, *P* = 0.7825; stimulation and treatment effect F (2, 12) = 352.4, *P <* 0.0001;

Ligp1: Interaction F (2, 12) = 0.4238, *P* = 0.664; inhibitor effect F (1, 12) = 0.839, *P* = 0.3777; stimulation and treatment effect F (2, 12) = 514.8, *P <* 0.0001;

Psmb8: Interaction F (2, 12) = 0.1807, *P* = 0.8369; inhibitor effect F (1, 12) = 0.05007, *P* = 0.8267; stimulation and treatment effect F (2, 12) = 368.4, *P <* 0.0001;

Srgn: Interaction F (2, 12) = 0.08105, *P* = 0.9227; inhibitor effect F (1, 12) = 0.02609, *P* = 0.8744; stimulation and treatment effect F (2, 12) = 85.7, *P <* 0.0001;

Amigo2: Interaction F (2, 12) = 1.145, *P* = 0.3506; inhibitor effect F (1, 12) = 0.09077, *P* = 0.7684; stimulation and treatment effect F (2, 12) = 126.3, *P <* 0.0001;

C3: Interaction F (2, 12) = 1.578, *P* = 0.2463; inhibitor effect F (1, 12) = 0.03915, *P* = 0.8465; stimulation and treatment effect F (2, 12) = 2599, *P <* 0.0001;

**B**: Serping1: Interaction F (2, 12) = 2.373, *P* = 0.1354; inhibitor effect F (1, 12) = 0.7828, *P* = 0.3937; stimulation and treatment effect F (2, 12) = 83.04, *P <* 0.0001;

Ligp1: Interaction F (2, 12) = 3.315, *P* = 0.0714; inhibitor effect F (1, 12) = 0.1478, *P* = 0.7074; stimulation and treatment effect F (2, 12) = 151.3, *P <* 0.0001;

Psmb8: Interaction F (2, 12) = 0.3112, *P* = 0.7383; inhibitor effect F (1, 12) = 0.1255, *P* = 0.7293; stimulation and treatment effect F (2, 12) = 39.47, *P <* 0.0001;

Srgn: Interaction F (2, 12) = 0.4793, *P* = 0.6306; inhibitor effect F (1, 12) = 0.2536, *P* = 0.6237; stimulation and treatment effect F (2, 12) = 78.37, *P <* 0.0001;

Amigo2: Interaction F (2, 12) = 3.542, *P* = 0.0618; inhibitor effect F (1, 12) = 0.9311, *P* = 0.3536; stimulation and treatment effect F (2, 12) = 52.42, *P <* 0.0001;

C3: Interaction F (2, 12) = 0.5159, *P* = 0.6096; inhibitor effect F (1, 12) = 0.3083, *P* = 0.5889; stimulation and treatment effect F (2, 12) = 82.37, *P <* 0.0001.

**Fig. 6**

**A**: Serping1: Interaction F (6, 24) = 21.7, *P <* 0.0001; stimulation and treatment effect F (3, 24) = 202.2, *P <* 0.0001; genotype effect F (2, 24) = 50.67, *P <* 0.0001;

Ligp1: Interaction F (6, 24) = 22.43, *P <* 0.0001; stimulation and treatment effect F (3, 24) = 311.3, *P <* 0.0001; genotype effect F (2, 24) = 30.3, *P <* 0.0001;

Psmb8: Interaction F (6, 24) = 34.78, *P <* 0.0001; stimulation and treatment effect F (3, 24) = 567, *P <* 0.0001; genotype effect F (2, 24) = 49.95, *P <* 0.0001;

Srgn: Interaction F (6, 24) = 19.97, *P <* 0.0001; stimulation and treatment effect F (3, 24) = 219.4, *P <* 0.0001; genotype effect F (2, 24) = 39.35, *P <* 0.0001;

Amigo2: Interaction F (6, 24) = 14.39, *P* = 0.0507; stimulation and treatment effect F (3, 24) = 53.94, *P* = 0.0661; genotype effect F (2, 24) = 34.31, *P <* 0.0001;

C3: Interaction F (6, 24) = 59.33, *P <* 0.0001; stimulation and treatment effect F (3, 24) = 1124, *P <* 0.0001; genotype effect F (2, 24) = 62.76, *P <* 0.0001;

**B**: Serping1: Interaction F (6, 24) = 75.93, *P <* 0.0001; stimulation and treatment effect F (3, 24) = 705.6, *P <* 0.0001; genotype effect F (2, 24) = 214.1, *P <* 0.0001;

Ligp1: Interaction F (6, 24) = 24.7, *P <* 0.0001; stimulation and treatment effect F (3, 24) = 305.2, *P <* 0.0001; genotype effect F (2, 24) = 28.9, *P <* 0.0001;

Psmb8: Interaction F (6, 24) = 4.855, *P* = 0.0022; stimulation and treatment effect F (3, 24) = 70.32, *P <* 0.0001; genotype effect F (2, 24) = 1.803, *P* = 0.1865;

Srgn: Interaction F (6, 24) = 13.52, *P <* 0.0001; stimulation and treatment effect F (3, 24) = 114.3, *P <* 0.0001; genotype effect F (2, 24) = 6.861, *P* = 0.0044;

Amigo2: Interaction F (6, 24) = 20.01, *P <* 0.0001; stimulation and treatment effect F (3, 24) = 180.4, *P <* 0.0001; genotype effect F (2, 24) = 69.95, *P <* 0.0001;

C3: Interaction F (6, 24) = 16.52, *P <* 0.0001; stimulation and treatment effect F (3, 24) = 73.75, *P <* 0.0001; genotype effect F (2, 24) = 21.78, *P <* 0.0001.

**Fig. 7**

**A**: Interaction F (70, 621) = 5.482, *P <* 0.0001; time effect F (10, 621) = 17.54, *P <* 0.0001; stimulation and treatment effect F (7, 621) = 124.2, *P <* 0.0001;

**B-TST**: Interaction F (3, 64) = 3.671, *P* = 0.0166; stimulation and treatment effect F (3, 64) = 34.42, *P <* 0.0001; genotype effect F (1, 64) = 3.824, *P* = 0.0549;

**B-FST**: Interaction F (3, 60) = 5.063, *P* = 0.0034; stimulation and treatment effect F (3, 60) = 62.97, *P <* 0.0001; genotype effect F (1, 60) = 0.1258, *P* = 0.7241;

**D**: Interaction F (3, 24) = 77.59, *P <* 0.0001; stimulation and treatment effect F (3, 24) = 342.2, *P <* 0.0001; genotype effect F (1, 24) = 79.81, *P <* 0.0001;

**F**: Interaction F (3, 24) = 9.535, *P* = 0.0002; stimulation and treatment effect F (3, 24) = 46.98, *P <* 0.0001; genotype effect F (1, 24) = 12.43, *P* = 0.0017;

**G**: Serping1: Interaction F (3, 24) = 19.56, *P <* 0.0001; stimulation and treatment effect F (3, 24) = 150.1, *P <* 0.0001; genotype effect F (1, 24) = 20.23, *P* = 0.0001;

Ligp1: Interaction F (3, 24) = 140.9, *P <* 0.0001; stimulation and treatment effect F (3, 24) = 654.3, *P <* 0.0001; genotype effect F (1, 24) = 149.8, *P <* 0.0001;

Psmb8: Interaction F (3, 24) = 74.74, *P <* 0.0001; stimulation and treatment effect F (3, 24) = 263.5, *P <* 0.0001; genotype effect F (1, 24) = 24.76, *P <* 0.0001;

Amigo2: Interaction F (3, 24) = 18.87, *P <* 0.0001; stimulation and treatment effect F (3, 24) = 80.72, *P <* 0.0001; genotype effect F (1, 24) = 26.03, *P <* 0.0001;

C3: Interaction F (3, 24) = 74.74, *P <* 0.0001; stimulation and treatment effect F (3, 24) = 263.5, *P <* 0.0001; genotype effect F (1, 24) = 24.76, *P <* 0.0001.

**Fig. S1**

**C**: Interaction F (1, 28) = 8.817, *P* = 0.0061; treatment effect F (1, 28) = 5.183, *P* = 0.0307; stimulation effect F (1, 28) = 5.643, *P* = 0.0246;

**D**: Interaction F (1, 28) = 3.58, *P* = 0.0689; treatment effect F (1, 28) = 4.364, *P* = 0.0459; stimulation effect F (1, 28) = 4.121;

**E**: Interaction F (1, 28) = 11.57, *P* = 0.002; treatment effect F (1, 28) = 10.39, *P* = 0.0032; stimulation effect F (1, 28) = 10.59, *P* = 0.003;

**F**: Interaction F (1, 28) = 7.341, *P* = 0.0114; treatment effect F (1, 28) = 3.997, *P* = 0.0554; stimulation effect F (1, 28) = 17.63, *P* = 0.0002;

**Fig. S2**

**A**: H2-T23: Interaction F (1, 12) = 0.462, *P* = 0.5096; treatment effect F (1, 12) = 0.1183, *P* = 0.7368; stimulation effect F (1, 12) = 5.746, *P* = 0.0337;

Serping1: Interaction F (1, 12) = 31.07, *P* = 0.0001; treatment effect F (1, 12) = 36.99, *P <* 0.0001; stimulation effect F (1, 12) = 30.62, *P* = 0.0001;

H2-D1: Interaction F (1, 12) = 45.47, *P <* 0.0001; treatment effect F (1, 12) = 0.03357, *P* = 0.8577; stimulation effect F (1, 12) = 9.634, *P* = 0.0091;

Ggta1: Interaction F (1, 12) = 9.892, *P* = 0.0084; treatment effect F (1, 12) = 1.272, *P* = 0.2814; stimulation effect F (1, 12) = 3.939, *P* = 0.0705;

Ligp1: Interaction F (1, 12) = 43.5, *P <* 0.0001; treatment effect F (1, 12) = 66.41, *P <* 0.0001; stimulation effect F (1, 12) = 89.85, *P <* 0.0001;

Gbp2: Interaction F (1, 12) = 0.8262, *P* = 0.3813; treatment effect F (1, 12) = 0.288, *P* = 0.6013; stimulation effect F (1, 12) = 0.8559, *P* = 0.3731;

Fbln5: Interaction F (1, 12) = 0.2203, *P* = 0.6472; treatment effect F (1, 12) = 0.01567, *P* = 0.9025; stimulation effect F (1, 12) = 0.000998, *P* = 0.9753;

Ugt1a: Interaction F (1, 12) = 0.2732, *P* = 0.6107; treatment effect F (1, 12) = 0.1493, *P* = 0.7059; stimulation effect F (1, 12) = 1.407, *P* = 0.2584;

Fkbp5: Interaction F (1, 12) = 0.3999, *P* = 0.539; treatment effect F (1, 12) = 0.01013, *P* = 0.9215; stimulation effect F (1, 12) = 3.401, *P* = 0.09;

Psmb8: Interaction F (1, 12) = 89.08, *P <* 0.0001; treatment effect F (1, 12) = 83.01, *P <* 0.0001; stimulation effect F (1, 12) = 68.18, *P <* 0.0001;

Srgn: Interaction F (1, 12) = 1.142, *P* = 0.3063; treatment effect F (1, 12) = 0.5136, *P* = 0.4873; stimulation effect F (1, 12) = 24.74, *P* = 0.0003;

Amigo2: Interaction F (1, 12) = 108.8, *P <* 0.0001; treatment effect F (1, 12) = 112.9, *P <* 0.0001; stimulation effect F (1, 12) = 170.2, *P <* 0.0001;

C3: Interaction F (1, 12) = 248.8, *P <* 0.0001; treatment effect F (1, 12) = 273.5, *P <* 0.0001; stimulation effect F (1, 12) = 188.9, *P <* 0.0001;

Clcf1: Interaction F (1, 12) = 4.104, *P* = 0.0656; treatment effect F (1, 12) = 6.478, *P* = 0.0257; stimulation effect F (1, 12) = 11.45, *P* = 0.0054;

Ptx3: Interaction F (1, 12) = 0.4159, *P* = 0.5311; treatment effect F (1, 12) = 1.008, *P* = 0.3352; stimulation effect F (1, 12) = 2.181, *P* = 0.1655;

S100a10: Interaction F (1, 12) = 2.479, *P* = 0.1413; treatment effect F (1, 12) = 0.47, *P* = 0.506; stimulation effect F (1, 12) = 2.194, *P* = 0.1643;

Sphk1: Interaction F (1, 12) = 0.933, *P* = 0.3531; treatment effect F (1, 12) = 0.004098, *P* = 0.95; stimulation effect F (1, 12) = 1.725, *P* = 0.2136;

Cd109: Interaction F (1, 12) = 2.453, *P* = 0.1433; treatment effect F (1, 12) = 0.008347, *P* = 0.9287; stimulation effect F (1, 12) = 0.7203, *P* = 0.4127;

Ptgs2: Interaction F (1, 12) = 1.332, *P* = 0.2709; treatment effect F (1, 12) = 1.691, *P* = 0.2179; stimulation effect F (1, 12) = 1.899, *P* = 0.1933;

Emp1: Interaction F (1, 12) = 1.501, *P* = 0.244; treatment effect F (1, 12) = 1.153, *P* = 0.304; stimulation effect F (1, 12) = 0.08712, *P* = 0.7729;

Slc10a6: Interaction F (1, 12) = 0.7397, *P* = 0.4066; treatment effect F (1, 12) = 1.937, *P* = 0.1892; stimulation effect F (1, 12) = 1.44, *P* = 0.2532;

Tm4sf1: Interaction F (1, 12) = 0.06281, *P* = 0.8063; treatment effect F (1, 12) = 0.02336, *P* = 0.8811; stimulation effect F (1, 12) = 0.4147, *P* = 0.5317;

B3gnt5: Interaction F (1, 12) = 1.545, *P* = 0.2377; treatment effect F (1, 12) = 0.07492, *P* = 0.789; stimulation effect F (1, 12) = 1.786, *P* = 0.2062;

Cd14: Interaction F (1, 12) = 0.7225, *P* = 0.412; treatment effect F (1, 12) = 1.647, *P* = 0.2236; stimulation effect F (1, 12) = 0.09372, *P* = 0.7647

**C**: Interaction F (1, 12) = 27.16, *P* = 0.0002; treatment effect F (1, 12) = 10.09, *P* = 0.008; stimulation effect F (1, 12) = 4.285, *P* = 0.0607;

**D**: serum: Interaction F (1, 12) = 515, *P <* 0.0001; treatment effect F (1, 12) = 5195, *P <* 0.0001; stimulation effect F (1, 12) = 5333, *P <* 0.0001;

cortex: Interaction F (1, 12) = 53.84, *P <* 0.0001; treatment effect F (1, 12) = 16.37, *P* = 0.0016; stimulation effect F (1, 12) = 89.69, *P <* 0.0001;

hippocampus: Interaction F (1, 12) = 69.39, *P <* 0.0001; treatment effect F (1, 12) = 66.47, *P <* 0.0001; stimulation effect F (1, 12) = 54.17, *P <* 0.0001.

**Fig. S3**

**B**: Interaction F (1, 8) = 32.01, *P* = 0.0005; treatment effect F (1, 8) = 179.2, *P <* 0.0001; stimulation effect F (1, 8) = 12.9, *P* = 0.0071;

**C**: Interaction F (1, 8) = 13.68, *P* = 0.0061; treatment effect F (1, 8) = 58.24, *P <* 0.0001; stimulation effect F (1, 8) = 1.845, *P* = 0.2115;

**D**: Interaction F (1, 8) = 34.65, *P* = 0.0004; treatment effect F (1, 8) = 42.46, *P* = 0.0002; stimulation effect F (1, 8) = 7.408, *P* = 0.0262.

**Fig. S6**

**B**: Interaction F (3, 61) = 4.663, *P* = 0.0053; stimulation and treatment effect F (3, 61) = 20.07, *P <* 0.0001; AAV effect F (1, 61) = 2.086, *P* = 0.1537;

**C**: Interaction F (3, 61) = 4.171, *P* = 0.0094; stimulation and treatment effect F (3, 61) = 19.43, *P <* 0.0001; AAV effect F (1, 61) = 3.367, *P* = 0.0714;

**D**: Interaction F (3, 60) = 6.831, *P* = 0.0005; stimulation and treatment effect F (3, 60) = 23.31, *P <* 0.0001; AAV effect F (1, 60) = 10.43, *P* = 0.002;

**E**: Interaction F (3, 56) = 4.625, *P* = 0.0058; stimulation and treatment effect F (3, 56) = 15.43, *P <* 0.0001; AAV effect F (1, 56) = 6.021, *P* = 0.0173;

**F**: Serping1: Interaction F (3, 24) = 45.43, *P <* 0.0001; stimulation and treatment effect F (3, 24) = 205.7, *P <* 0.0001; AAV effect F (1, 24) = 42.63, *P <* 0.0001;

Ligp1: Interaction F (3, 24) = 256.7, *P <* 0.0001; stimulation and treatment effect F (3, 24) = 930.3, *P <* 0.0001; AAV effect F (1, 24) = 263.4, *P <* 0.0001;

Psmb8: Interaction F (3, 24) = 68.52, *P <* 0.0001; stimulation and treatment effect F (3, 24) = 328.1, *P <* 0.0001; AAV effect F (1, 24) = 76.57, *P <* 0.0001;

Amigo2: Interaction F (3, 24) = 114.9, *P <* 0.0001; stimulation and treatment effect F (3, 24) = 389.5, *P <* 0.0001; AAV effect F (1, 24) = 110.6, *P <* 0.0001;

C3: Interaction F (3, 24) = 34.58, *P <* 0.0001; stimulation and treatment effect F (3, 24) = 123.2, *P <* 0.0001; AAV effect F (1, 24) = 44.49, *P <* 0.0001.

**Fig. S7**

**C**: Serping1: Interaction F (3, 16) = 33.49, *P <* 0.0001; stimulation and treatment effect F (3, 16) = 683.4, *P <* 0.0001; genotype effect F (1, 16) = 13.48, *P* = 0.0021;

Ligp1: Interaction F (3, 16) = 8.661, *P* = 0.0012; stimulation and treatment effect F (3, 16) = 332, *P <* 0.0001; genotype effect F (1, 16) = 9.117, *P* = 0.0081;

Psmb8: Interaction F (3, 16) = 11.71, *P* = 0.0003; stimulation and treatment effect F (3, 16) = 434.9, *P <* 0.0001; genotype effect F (1, 16) = 23.79, *P* = 0.0002;

Srgn: Interaction F (3, 16) = 26.55, *P <* 0.0001; stimulation and treatment effect F (3, 16) = 166.8, *P <* 0.0001; genotype effect F (1, 16) = 105.7, *P <* 0.0001;

Amigo2: Interaction F (3, 16) = 8.564, *P* = 0.0013; stimulation and treatment effect F (3, 16) = 33.69, *P <* 0.0001; genotype effect F (1, 16) = 5.436, *P* = 0.0331;

C3: Interaction F (3, 16) = 6.741, *P* = 0.0038; stimulation and treatment effect F (3, 16) = 259.8, *P <* 0.0001; genotype effect F (1, 16) = 12.34, *P* = 0.0029.

**Fig. S8**

**B**: Interaction F (3, 63) = 9.63, *P <* 0.0001; stimulation and treatment effect F (3, 63) = 44.06, *P <* 0.0001; genotype effect F (1, 63) = 6.014, *P* = 0.017;

**C**: Interaction F (3, 63) = 3.86, *P* = 0.0134; stimulation and treatment effect F (3, 63) = 34.19, *P <* 0.0001; genotype effect F (1, 63) = 2.391, *P* = 0.127;

**D**: Interaction F (3, 58) = 6.527, *P* = 0.0007; stimulation and treatment effect F (3, 58) = 36.47, *P <* 0.0001; genotype effect F (1, 58) = 2.641, *P* = 0.1095;

**E**: Interaction F (3, 56) = 7.197, *P* = 0.0004; stimulation and treatment effect F (3, 56) = 68.27, *P <* 0.0001; genotype effect F (1, 56) = 2.29, *P* = 0.1359;

**F**: Serping1: Interaction F (3, 24) = 40.57, *P <* 0.0001; stimulation and treatment effect F (3, 24) = 147.1, *P <* 0.0001; genotype effect F (1, 24) = 60.26, *P <* 0.0001;

Ligp1: Interaction F (3, 24) = 202.8, *P <* 0.0001; stimulation and treatment effect F (3, 24) = 686.3, *P <* 0.0001; genotype effect F (1, 24) = 183.2, *P <* 0.0001;

Psmb8: Interaction F (3, 24) = 197.6, *P <* 0.0001; stimulation and treatment effect F (3, 24) = 913.6, *P <* 0.0001; genotype effect F (1, 24) = 214.1, *P <* 0.0001;

Amigo2: Interaction F (3, 24) = 245.1, *P <* 0.0001; stimulation and treatment effect F (3, 24) = 796.8, *P <* 0.0001; genotype effect F (1, 24) = 193.2, *P <* 0.0001;

C3: Interaction F (3, 24) = 31.48, *P <* 0.0001; stimulation and treatment effect F (3, 24) = 133.9, *P <* 0.0001; genotype effect F (1, 24) = 48.09, *P <* 0.0001.
